# Zika Virus and Arthritis/Arthralgia: A Systematic Review and Meta-Analysis

**DOI:** 10.3390/v12101137

**Published:** 2020-10-07

**Authors:** B.M.C.R. Wimalasiri-Yapa, Harith E. Yapa, Xiaodong Huang, Louise M. Hafner, Tony J. Kenna, Francesca D. Frentiu

**Affiliations:** 1 School of Biomedical Sciences, Faculty of Health, Queensland University of Technology, Brisbane QLD 4000, Australia; badal.yapa@hdr.qut.edu.au (B.M.C.R.W.-Y.); x1.huang@qut.edu.au (X.H.); l.hafner@qut.edu.au (L.M.H.); tony.kenna@qut.edu.au (T.J.K.); 2 Department of Medical Laboratory Sciences, Faculty of Health Sciences, The Open University of Sri Lanka, Colombo 10250, Sri Lanka; 3 School of Nursing, Faculty of Health, Queensland University of Technology, Brisbane QLD 4059, Australia; haritheranga.yapa@hdr.qut.edu.au; 4 Department of Nursing, Faculty of Health Sciences, The Open University of Sri Lanka, Colombo 10250, Sri Lanka

**Keywords:** zika virus, arthralgia, arthritis, rheumatic, joint

## Abstract

Dengue, chikungunya and Zika viruses share similar disease features, rendering them difficult to distinguish clinically. Incapacitating arthralgia/arthritis is a specific manifestation associated with chikungunya virus infection. However, the profile of arthralgia/arthritis in Zika virus (ZIKV) cases has not been well characterized. Articles were extracted from PubMed and Scopus databases reporting original data from patients with arthralgia/arthritis, according to the Cochrane Collaboration. Following Preferred Reporting Items for Systematic Reviews and Meta-Analyses guidelines, 137 articles reporting ZIKV-associated joint symptoms were reviewed. Arthralgia was more frequently reported (*n* = 124 from case studies, *n* = 1779 from population-based studies) than arthritis (*n* = 7 and *n* = 121, respectively). Arthralgia was resolved in <1 week in 54%, and within 1–2 weeks in 40% of cases. The meta-analysis of cases in population-based studies identified a pooled prevalence of 53.55% for arthralgia. The pooled prevalence of arthralgia/arthritis during outbreaks depended on the geographic location, with a higher joint symptom burden observed in the Americas compared to South East Asia (Brazil: 60.79%; Puerto Rico: 68.89% and South East Asia: 26.46%). We conclude that non-specific constitutional arthralgia is the most common joint manifestation during ZIKV infection, being present in nearly half of cases but resolving by two weeks in >90% of these. We found no evidence of chronic rheumatic manifestations following ZIKV infection.

## 1. Introduction

**Figure 1:**
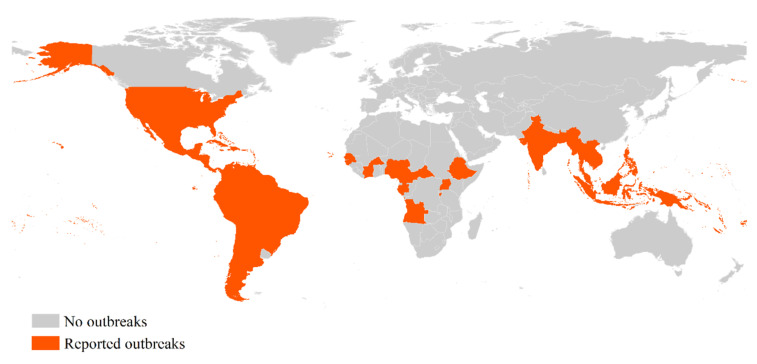
Countries and territories with current or previous Zika virus autochthonous transmission, by the WHO regional office (data as at 2 July 2019).

Zika virus (ZIKV) is an emerging arbovirus that belongs to the *Flavivirus* genus in the *Flaviviridae* family [1]. Following detection of ZIKV from a rhesus monkey in 1947, human infections were first reported in Nigeria in 1952 [2]. Until 2007, documented ZIKV circulation was limited to Asia and Africa but without reports of major outbreaks [3]. In 2007, a large ZIKV outbreak was reported from Yap island of the Federated States of Micronesia [4]. Duffy et al. (2009) estimated that 73% of Yap residents three years of age or older were infected during this outbreak [4]. Then in 2013, French Polynesia experienced a ZIKV outbreak where virus-associated neurological disease was reported for the first time [5]. Serosurveys conducted 18 months post-outbreak in French Polynesia showed a ZIKV seroprevalence rate of 49% [6]. The virus then reached the Americas in 2015, and local transmission was identified in 20 countries by mid-January 2016 [7]. Thereafter, ZIKV was declared a global public health emergency of international concern in 2016 by the World Health Organization (WHO), after causing massive outbreaks with neurological defects resulting from the disease [8]. To date, there are 87 countries that have reported autochthonous transmission of ZIKV (see Figure 1) [9]. Reports from the year 2020 indicate ZIKV cases in Vietnam, Brazil and Puerto Rico, suggestive of active circulation of the virus in the Americas and Asia [10]. While a considerable proportion of cases were asymptomatic [7,11], the common symptoms of the disease include fever, rash, conjunctivitis, muscle and joint pain, malaise or headache [12]. Due to the increased risk of neurologic complications associated with ZIKV infection, it is crucial to differentiate this disease from other febrile, mosquito-borne viruses such as dengue and chikungunya. ZIKV shares many of the same clinical features of above-mentioned diseases, all of which are transmitted by the same vector, predominantly *Aedes aegypti* mosquitoes [13]. Moreover, the ability of ZIKV to be sexually transmitted by human travelers to partners who have not been to places where the virus is endemic and vertical transmission from viremic mothers during delivery increases the risk of emergence of the disease in new areas [14].

Arthritis is defined as pain with swelling of the joints, while arthralgia often refers to joint pain without swelling [15]. Use of these terms, however, is frequently not well defined in many studies. Parvoviruses, alphaviruses such as chikungunya and Ross river viruses, hepatitis B virus, hepatitis C virus, Epstein-Barr virus (EBV) and Zika virus are all known to cause viral arthritis [16]. However, the exact incidence and prevalence of ZIKV arthritis/arthralgia is unknown to date. While the operational case definitions of chikungunya and Zika virus infections list arthralgia as a primary symptom [12,17], chikungunya is more widely acknowledged to cause incapacitating polyarthralgia or arthritis as the first symptom in the acute phase and persisting in the chronic phase [18]. However, due to limited detection or lack of reporting of the disease [19], the nature of ZIKV arthritis/arthralgia remains uncharacterised. In this paper, we systematically review the profile of ZIKV cases reporting joint symptoms/signs and conduct a meta-analysis to estimate the prevalence of arthritis/arthralgia in ZIKV-infected populations.

## 2. Materials and Methods 

The systematic review was performed according to the Cochrane Collaboration guidelines [20], and findings compiled following the “Preferred Reporting Items for Systematic Reviews and Meta-analyses” (PRISMA) format [21].

### 2.1. Search Strategy and Selection

The report type, study design and outcomes reported in each record were systematically screened for inclusion or exclusion in the review, based on predefined eligibility criteria (see Table 1). According to the WHO, a confirmed ZIKV case can be defined as a person with laboratory confirmation of recent Zika virus infection by: either presence of ZIKV RNA or antigen in serum or other samples (e.g., saliva, tissues, urine, whole blood); or presence of IgM antibody against ZIKV-positive and PRNT90 for ZIKV with titre ≥20 and ZIKV PRNT90 titre ratio ≥ 4 compared to other flaviviruses; and exclusion of other flaviviruses. All articles with confirmed ZIKV infections (according to WHO/Centers for Disease Control and Prevention or any other guidelines given by regional/local authorities), over any time interval and in human populations, were considered for inclusion in this study. PubMed and Scopus databases were searched by using the keywords: “Zika” and “arthritis” or “arthralgia” or “joint pain” or “joint swelling” or “rheumatology” or “inflammatory joint pain” (refer to Table 2 for complete search strategy). The search was conducted from 07th February 2019 to 12th July 2019 and updated on 12th August 2020.

### 2.2. Data Analysis

Records were imported into Endnote X8 bibliographic software (X8.0.1; Thompson Reuters, Philadelphia, USA) and a Microsoft Excel file. Duplicate publications were removed, and records containing the same research data/findings published by the same author in different formats/titles were counted only once. Retrieved titles and abstracts, and then potentially eligible full-text records, were screened based on the predefined inclusion and exclusion criteria (see Table 2). Records required an abstract in the English language to be included. Ineligible records were excluded from analyses, and eligible reports were used for data extraction. The following data were extracted and recorded in tables: study/report type (surveillance/serological survey, imported case, case study, sporadic case, case series, disease cluster, outbreak or epidemic), demography (age and sex), year and place. Age and sex were reported as “inconclusive” when the individual case information is not clearly mentioned.

Basic descriptive analysis of the data was performed using GraphPad Prism version 7 (GraphPad Software, La Jolla, California, USA). A meta-analysis was performed to calculate the prevalence of joint symptoms among confirmed ZIKV cases. The proportion of cases with joint symptoms in each study was combined to give a pooled prevalence of joint symptoms in ZIKV infections. All analyses were performed with Review Manager 5.4 [22]. Heterogeneity was assessed using the I^2^ statistic and Chi-square test (I^2^ value of 0–25, 26–50, 51–75 and 75–100% indicates low, moderate, high and very high heterogeneity, and *p*-value < 0.10 defines a statistically significant degree) [20]. A random-effects model was used for where a high level of heterogeneity was observed. Non-overlap of the confidence intervals indicated statistical significance when there were only two subgroups present in the meta-analysis [20]. 

## 3. Results

**Figure 2:**
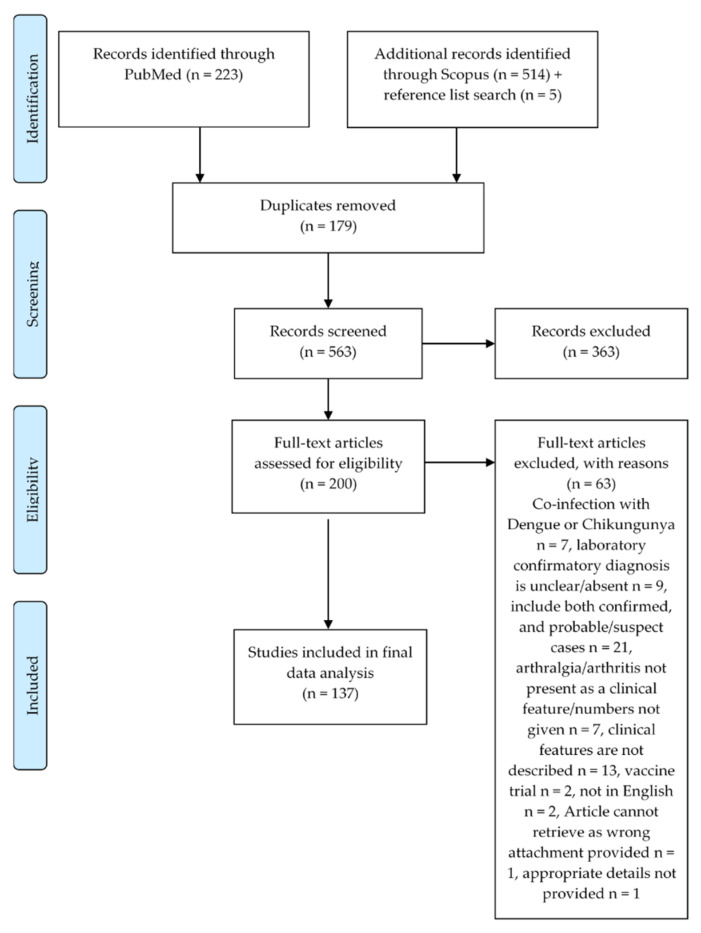
Preferred Reporting Items for Systematic Reviews and Meta-Analyses (PRISMA) flow chart of included studies.

We identified 742 articles, from which 179 duplicates were removed (see Figure 2). Among the remaining 563 articles, 363 were excluded during the screening of abstracts as they did not meet the eligibility criteria. The full texts of the remaining 200 articles then were assessed for eligibility for inclusion in the current study. Following the full-text review, 63 records were excluded according to the criteria given in Figure 1. Therefore, a total of 137 articles were considered for further analysis in this study (see Supplementary Tables S1 and S2). Among these 137 articles, there were case studies, epidemiological studies, screening or surveillance studies, observational studies, cross-sectional studies, case-control studies and cohort studies. Altogether, there were 4783 cases of confirmed ZIKV infections in people who presented with symptomatic diseases.

### 3.1. Regional Distribution of Records Reporting ZIKV with Joint Signs/Symptoms

According to the geographic regions classified by the WHO, the majority of records reporting ZIKV with joint signs/symptoms were from the Americas (*n* = 96), followed by European region (*n* = 27) countries, South East Asia (*n* = 8), the Western Pacific region (*n* = 5) and one article from Africa. There were 41 countries identified that had reports of confirmed ZIKV infection with joint signs/symptoms. The highest number of countries was in the Americas, involving 17 countries. ZIKV-associated joint signs/symptoms were also reported from 13 European countries, six South East Asian countries, four Western Pacific region countries, and one African country (see Table 3). All 27 articles describing cases reported in European countries concerned imported ZIKV infections (see Supplementary Table S1).

### 3.2. Outbreaks of ZIKV with Joint Signs/Symptoms

Thirteen countries reported outbreaks of ZIKV with joint signs/symptoms, including an epidemic in Mexico. The majority of the papers identified described the 2015–2016 Brazil ZIKV outbreak (29 articles including 12 case reports and 17 population-based study reports). The other outbreaks occurred in: Suriname (2015–2016), Puerto Rico (2016), Panama (2015–2016), Peru (2016), Mexico (October 2015–August 2017), Martinique (2015–2016), Honduras (2015–2016), Colombia (2015–2016), Dominican Republic (mentioned as an epidemic, 2016–2017), French Polynesia (2013–2014), Micronesia (2017) and Thailand (2016). Cases from Europe only indicated the detection of travel-related (imported and/or sexually transmitted to the non-travelled partner) disease.

### 3.3. Case Studies Reporting Confirmed ZIKV Cases with Joint Signs/Symptoms

**Figure 3:**
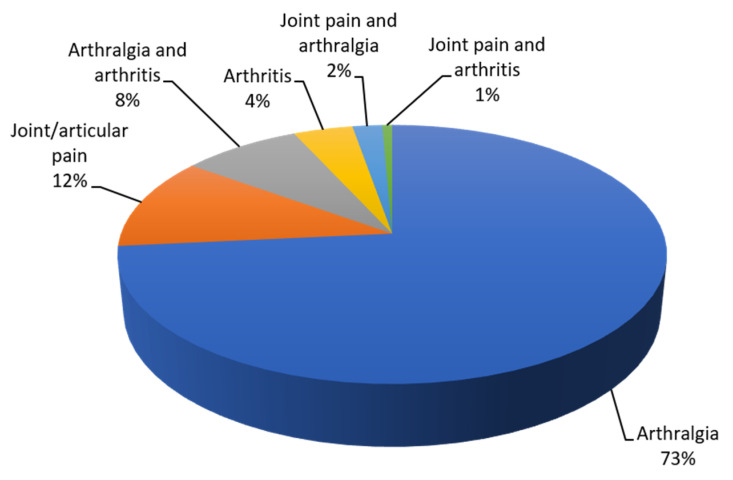
Terminologies used in case studies to describe joint signs/symptoms (% of total cases).

There was a total of 78 articles describing 251 confirmed ZIKV-infected cases (see Supplementary Table S1). Among these, 147 cases described joint symptoms and these symptoms were reported using different terminologies including arthralgia, joint pain, arthritis (including joint swelling and oedema) and combinations of these three terms. The majority (*n* = 108, 73.47%) used the term arthralgia (see Figure 3).

Among 148 confirmed ZIKV cases with joint signs/symptoms, 106 reported the exact age of the persons. The cases ranged in ages from 15 to 80 years, with a median age of 43 years. There were another 10 cases that reported age information in decadal brackets. Those studies included five cases in their 20s, two cases in their 30s, and one patient each in their 40s, 50s and 70s. One study mentioned an adolescent and another described a young adult. The age information of the remaining 28 cases either was not reported or was reported as a summary measure (such as median) considering all the participants. The cases reporting arthritic signs/symptoms were mostly female, counting for 66.7 % of the 129 cases’ reported gender information. There was no information reported for 19 out of 148 patients. Information about comorbidities was documented in 36 articles regarding 42 confirmed ZIKV cases. The majority of cases (*n* = 29) were previously healthy or had an unremarkable medical history. The comorbidities of the remaining 13 cases are listed below in Box 1.

Box 1 **Past medical history reported for 13 confirmed ZIKV cases with joint manifestations.**

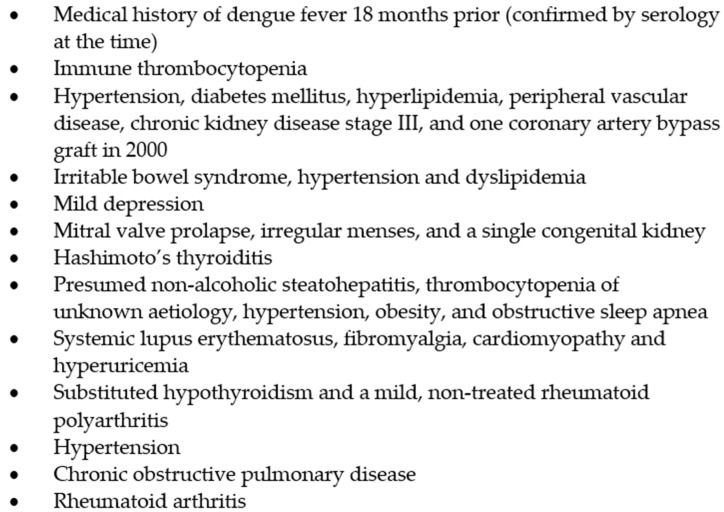

#### 3.3.1. Arthralgia and Joint/Articular Pain

**Figure 4:**
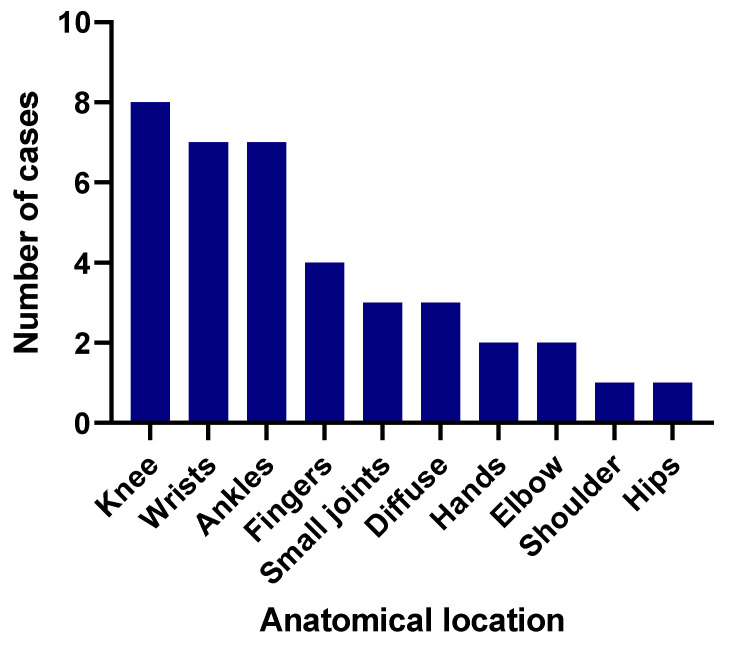
Anatomical location (x-axis) of joints affected by ZIKV-related arthralgia/joint pain, as reported in case studies (*n* = 19 articles and *n* = 38 cases).

We found (*n* = 50) patients for whom the exact duration of arthralgia was recorded (refer to Supplementary Table S1 for details). The mean duration was 6.98 days, with a range from one to 45 days. More than half of these cases (*n* = 27) indicated that arthralgia was resolved in less than a week, 40% indicated the symptoms lasted for one to two weeks, 4% for three weeks and only 2% reported the duration was >3 weeks. One patient reported that arthralgia recurred in two weeks after resolving initial symptoms. In addition, two cases indicated arthralgia of >11 days. The duration of the remaining 87 cases was not given. When considering the articles describing arthralgia, only 24 patient reports (in 19 articles) mentioned which exact joint was affected. Except for one patient with knee pain, all others described multiple joint involvement. Wrists (*n* = 9) and knees (*n* = 8) were the most affected among reported joints (see Figure 4).

#### 3.3.2. Arthritis

Arthritis/joint swelling/joint edema terms were used interchangeably by the authors of 14 articles that reported ZIKV-associated arthritis (see Table 4). Seven articles illustrated cases (*n* = 10) identified on examination by a clinician. Patient-reported arthritis (joint pain with swelling) was reported in seven articles describing eight cases. The most commonly affected joints were finger joints (*n* = 7), followed by ankles (*n* = 6), wrists (*n* = 4), knees (*n* = 2), elbows (*n* = 1), toes (*n* = 1), peripheral arthritis (*n* = 1) and three cases without specifying the affected joint. Except for six cases (including three that did not report the affected joint/s), all others reported multiple joint involvement. Six out of 18 cases reported previous medical history, among which three patients had an unremarkable medical history. Two patients reported a history of hypertension, and one hypertensive person also indicated irritable bowel syndrome and dyslipidemia. A third case provided a history of hypothyroidism and non-treated rheumatoid polyarthritis. The duration of arthritis ranged from two days to five weeks among nine cases who reported the time period. The mean and median duration was 10 days.

### 3.4. Population-Based Studies Reporting Confirmed ZIKV Cases with Joint Signs/Symptoms 

In addition to the case studies analysed there were 59 additional studies describing ZIKV occurrences by means of other study designs (Supplementary Table S2). These studies included eight epidemiological studies, 17 cohort studies (including both retrospective and prospective), 20 screening and surveillance studies, eight cross-sectional studies, two case-control studies and four hospital-based studies. Altogether, the 59 studies analysed in this review comprised 4702 confirmed ZIKV patients, among which 4530 cases reported clinical features including joint signs/symptoms. The arthritic clinical features were reported using many terminologies including arthritis, joint swelling or joint oedema, joint pain/ache, arthralgia, arthralgia/myalgia, or a combination of the above terms. The highest numbers of cases were reported as arthralgia (*n* = 1538) followed by “arthralgia or arthritis or peri-articular oedema” (*n* = 290), joint pain/ache (*n* = 241), arthralgia/myalgia (*n* = 55) and arthritis including joint swelling or joint oedema (*n* = 121).

#### 3.4.1. Meta-Analysis for Pooled Prevalence of Joint Signs/Symptoms

Articles that reported both arthralgia and arthritis as two separate symptoms were subjected to a meta-analysis. There were nine studies with a moderate heterogeneity (I^2^ = 47%) when using a random-effect model where the odds ratio was 0.20 (95% CI: 0.13–0.31). The odds of arthritis in confirmed ZIKV cases were 80% less than confirmed ZIKV cases with arthralgia (see Supplementary Table S3). One eligible study (de Laval et al., 2018) was not included in the analysis as the article reported the symptoms at multiple time points.

A total of 40 articles only reporting arthralgia or joint pain was included in a meta-analysis to calculate the overall pooled prevalence of arthralgia among confirmed ZIKV cases. A very high level of heterogeneity was observed (I^2^ =  95%, *p*  <  0.001) among studies; therefore, a random-effects model was used to estimate the overall pooled prevalence of joint symptoms among confirmed ZIKV cases as 53.55 % (95%CI: 46.14–60.96) (see Supplementary Table S4). When considering all 59 articles, there was a very high heterogeneity among the studies (I^2^ = 93%). Analysis based on further subgroups of the population (study design, number of confirmed cases, immunity status: children or pregnant women) was not able to explain the heterogeneity observed (see Supplementary Tables S5–S7).

#### 3.4.2. Prevalence of Joint Signs/Symptoms during ZIKV Outbreaks Depends on the Geographic Location

Many of the 59 articles with non-case report study design did not describe the epidemiology of the ZIKV outbreaks. Therefore, we selected 13 articles out of these 59 that had epidemiological descriptions of the ZIKV outbreaks to use in a meta-analysis to identify the prevalence of joint symptoms among confirmed cases during an outbreak. The meta-analysis indicated that the prevalence of joint symptoms during an outbreak of ZIKV ranged from 22.8% to 79.8%. A very high level of heterogeneity was observed (I^2^ =  93%, *p*  <  0.001); therefore, a random effect model was used to estimate the overall pooled prevalence of joint symptoms among confirmed ZIKV cases during an outbreak (see Supplementary Table S8). Further subgroup analysis was performed to evaluate the reason for high heterogeneity among joint symptom prevalence. When subgrouping studies, it was evident that the prevalence of joint symptoms depended on the geographic location of the outbreak (see Supplementary Table S8). The Brazil outbreak during 2015–2017 had a pooled prevalence of 60.79% (95% CI: 60.79–65.88), while that of Puerto Rico in 2016 was 68.87% (95% CI: 60.65–77.08). The outbreaks in South East Asian region had a lower pooled prevalence 26.46% (95% CI: 20.07–32.86). The Brazil, Puerto Rico and SEA outbreaks reported I^2^ values of 33%, 0% and 46%, which indicated moderate, low and moderate levels of heterogeneity, respectively.

## 4. Discussion

Nearly 80% of ZIKV infections are asymptomatic [23,24] and the remaining 20% are usually accompanied by non-specific symptoms such as fever, rash, arthralgia, myalgia, conjunctivitis and headache [25]. ZIKV infection in humans is associated with neurological complications, such as Guillain–Barre Syndrome in adults [26,27] and microcephaly in neonates [5]. The female preponderance of ZIKV confirmed cases observed in our study could be partly explained by participant recruitment biased towards studying pregnant women, given the significant consequences of viral infection in-utero. The majority of the articles describing ZIKV infections with joint signs/symptoms were from the Americas. Higher disease burden in the Americas and lack of screening/surveillance in SEA and WPR could explain the differences in numbers of articles. Furthermore, reasons for the absence of large ZIKV outbreaks in Asia remain unclear [28]. 

In case study reports and population-based studies, arthralgia (described as joint pain/ache) cases were reported 15–20 times more frequently than arthritis (described as joint swelling or oedema) among confirmed ZIKV cases. This indicates that arthralgia, which is a patient-reported manifestation, is more prevalent than arthritis, which is diagnosed by clinician observations. The mean duration of arthralgia (7days) was lower than that of arthritis (10 days). However, these values should be interpreted cautiously as the duration of arthritis was reported in only six case studies whilst none of the population-based studies provided such information. One article indicated recurrent arthralgias and none of the articles analysed in this review reported cases of recurrent arthritis. In contrast, a study on arthralgia duration in chikungunya virus infections showed that recurrent joint pain was prevalent in 21.4% of the study group [29]. The majority of ZIKV cases reported in this study indicated that arthralgia was resolved within two weeks. However, this finding is in contrast to those from chikungunya cases where the duration of severe joint pain can last from six months [30] to 18 months [29], and sometimes up to three years [31] in infected individuals.

We identified a pooled overall prevalence of arthralgia in ZIKV cases of 53.55 %, using a meta-analysis of population-based studies with a random effects model. However, high heterogeneity between studies limited our confidence in this estimate. High heterogeneity of results could either be due to selection bias or be due to true heterogeneity of the studies. Further analysis based on articles reporting outbreaks indicated that the prevalence could be as high as 68.87% in some outbreaks, such as Puerto Rico. A previous study comparing the prevalence of joint signs/symptoms between dengue and chikungunya infections indicated a lower prevalence (44%) in the former than the latter (90%) virus [32]. Taken together, these findings indicate that joint symptom prevalence associated with ZIKV infection is higher than observed for dengue but lower than for chikungunya.

Virus infections are known to be important causes of idiopathic autoimmune rheumatic diseases [33]. For instance, in some individuals, chikungunya virus induces a chronic phase of arthropathy by activating innate and cognate responses, alongside activation of macrophages and monocytes [34]. Ross River virus, an alphavirus in the same serogroup as chikungunya, has also been identified to cause prolonged rheumatic manifestations among 80–100% of patients up to 3–6 months post-infection [35]. Our review did not identify evidence of a chronic phase of rheumatic manifestations and arthropathy associated with ZIKV infection. Therefore arthralgia/arthritis could be considered a non-specific constitutional symptom for this virus. The pattern observed in ZIKV is similar to that for dengue virus [36], where arthralgia/arthritis can be considered a non-specific constitutional symptom. Our review also highlights that consensus on defining and differentiating rheumatic features associated with virus infections, and particularly during outbreaks, would result in a more complete characterization of disease manifestations and aid in clinical management. Further research into all musculoskeletal aspects of ZIKV infection would also benefit clinical management, although this was beyond the scope of our review. 

## 5. Conclusions

Nearly half of the ZIKV cases included in this study indicated arthralgia/arthritis as a symptom of ZIKV infection. The highest burden of ZIKV-associated joint symptoms in infected individuals was reported in studies from the Americas and reported in more females than males with confirmed ZIKV infection. Joint symptoms/signs were reported from ZIKV-infected patients with ages ranging from 15 to 80 years. Polyarthralgias were apparent in the majority of cases reporting the anatomical location of affected joints. ZIKV-associated arthralgia affected both small and large joints similarly, while arthritis predominantly affected the small joints. The prevalence of joint symptoms/signs during ZIKV outbreaks differed according to geographical location. The prevalence and duration of arthralgia was higher than for arthritis in confirmed ZIKV cases. ZIKV-associated arthralgias appeared non-specific and constitutional in nature and were resolved within two weeks post onset, with no evidence of a prolonged chronic phase. 

## Figures and Tables

**Table 1:** Criteria for inclusion or exclusion of records for this systematic review.

	Included	Excluded
**Report type**	Abstracts and full-length articles, conference proceedings, case reports, letters, brief reports, short communications, correspondence and editorials with novel data. English abstracts of full papers published in other languages were too included.	Opinions, letters, editorials and correspondence without new data; similar data or findings published under different titles or in different formats; review articles, book chapters, animal research or perspectives without new data.
**Study design**	Any randomised or non-randomised design.	Modelled data, review data.
**Results** **Place and time of occurrence**	Symptomatic ZIKV incidence in any human population during any time interval.	
**Type of the occurrence**	Imported cases, sporadic cases, case series or disease clusters; outbreaks and epidemics case reports.	
**Person (number of cases, age and sex) of affected people**	Only laboratory-confirmed human cases.	Laboratory-confirmed cases of co-infection of ZIKV with other diseases/infections (e.g. Dengue and Chikungunya) Missing or unclear laboratory-based method; suspected/probable ZIKV cases.
**Meta-analysis**	Articles describing epidemiological features of ZIKV outbreaks. Comparison of prevalence of arthralgia and arthritis in the studies that gave information about both manifestations separately in the same study.	Purposes other than epidemiological evaluation of ZIKV or restricted to defined population.

**Table 2:** Systematic review search criteria.

PubMed Search	Scopus Search
Search: ((((((arthralgia) OR (“joint pain”)) OR (rheumatology)) OR (“joint swelling”)) OR (“inflammatory joint pain”)) OR (arthritis)) AND (zika) Sort by: Most Recent (((((((“arthralgia”[MeSH Terms] OR “arthralgia”[All Fields]) OR “arthralgias”[All Fields]) OR “joint pain”[All Fields]) OR ((“rheumatology”[MeSH Terms] OR “rheumatology”[All Fields]) OR “rheumatology s”[All Fields])) OR “joint swelling”[All Fields]) OR “inflammatory joint pain”[All Fields]) OR (((“arthritis”[MeSH Terms] OR “arthritis”[All Fields]) OR “arthritides”[All Fields]) OR “polyarthritides”[All Fields])) AND ((((((“zika virus”[MeSH Terms] OR (“zika”[All Fields] AND “virus”[All Fields])) OR “zika virus”[All Fields]) OR “zika”[All Fields]) OR “zika virus infection”[MeSH Terms]) OR ((“zika”[All Fields] AND “virus”[All Fields]) AND “infection”[All Fields])) OR “zika virus infection”[All Fields]) Translations arthralgia: “arthralgia”[MeSH Terms] OR “arthralgia”[All Fields] OR “arthralgias”[All Fields] rheumatology: “rheumatology”[MeSH Terms] OR “rheumatology”[All Fields] OR “rheumatology’s”[All Fields] arthritis: “arthritis”[MeSH Terms] OR “arthritis”[All Fields] OR “arthritides”[All Fields] OR “polyarthritides”[All Fields] zika: “zika virus”[MeSH Terms] OR (“zika”[All Fields] AND “virus”[All Fields]) OR “zika virus”[All Fields] OR “zika”[All Fields] OR “zika virus infection”[MeSH Terms] OR (“zika”[All Fields] AND “virus”[All Fields] AND “infection”[All Fields]) OR “zika virus infection”[All Fields]	TITLE-ABS-KEY (arthralgia OR arthritis OR “joint pain” OR rheumatology OR “joint swelling” OR “inflammatory joint pain” AND zika)

**Table 3:** Regional distribution of studies reporting Zika virus (ZIKV) infection with arthritis/arthralgia.

Region	Number of Articles	Countries Reporting Confirmed ZIKV Cases with Joint Symptoms
African Region	1	Ghana
Region of the Americas	96	Brazil, Canada, Colombia, Cuba, Dominican Republic, French Guiana, Honduras, Martinique, Mexico, Nicaragua, Panama, Peru, Puerto Rico, Suriname, Trinidad and Tobago, United States, Venezuela
European Region	27	Belgium, Croatia, France, Germany, Italy, Netherlands, Norway, Portugal, Russia, Spain, Switzerland, Turkey, United Kingdom
Eastern Mediterranean Region	0	
Western Pacific Region	5	French Polynesia, Micronesia, New Caledonia, New Zealand
South-East Asia Region	8	Indonesia, Japan, Korea, Singapore, Viet Nam Thailand

**Table 4:** Case studies reporting arthritis.

Authors	Sign/Symptom	Reporter	Duration (Days)	Number of Cases	Joint(s) Affected	Country of Report	Age (Years)	Sex	Comorbidities	Diagnosed by	Source
Bachiller-Luque et al., 2016	Arthralgia and synovitis	Clinician	5	1	Fingers, right knee, ankle and elbow	Spain	49	Male	Irritable bowel syndrome, hypertension, dyslipidemia	RT-PCR	Imported
Penot et al., 2017	Arthritis, joints pain and swelling	Clinician	>10 (all cases)	3	Case 2—ankles and phalanges. Case 3—bilateral and symmetrical wrists, ankles and phalanges. Case 4—bilateral knees	France	36, 30, 39	All are female	Patient 3- Previously healthy Patient 4- hypothyroidism and non-treated rheumatoid polyarthritis	RT-PCR	Imported
Fabrizius et al., 2016	Effusion with tenderness to palpation	Clinician		1	Finger joints	United States	44	Male	Previously healthy	RT-PCR	Imported
Kulkarni et al., 2016	Mild synovitis	Clinician		1	Metacarpophalangeal and proximal interphalangeal joints bilaterally	United States	42	Female	Previously healthy	IgM and Zika virus PRNT	Imported
Rozé et al., 2016	Peripheral arthritis	Clinician		1	Peripheral arthritis	Martinique	Late 70s	Not given		RT-PCR	Local
Nicastri et al., 2016	Bilateral wrists swelling	Clinician		1	Bilateral wrists	Italy	74	Male		RT-PCR, serology, neutralisation	Imported
Zammarchi et al., 2015	Arthralgia and ankle oedema	Patient		2	Ankle	Italy	Early30s	Male & Female		RT-PCR/ IgG/IgM	Imported
Harrower et al., 2016	Arthralgia and ankle oedema	Patient		1	Ankle	New Zealand	51	Male		RT-PCR	Imported
Arsuaga et al., 2016	Arthritis	Patient	7, 8	2	Ankles and wrists	Spain	53, 51	Male & Female		RT-PCR	Imported
Cavalcanti et al., 2017	Articular edema	Patient		1	Fingers and toes	Brazil	42	Female		RT-PCR	Local
Vinhaes et al., 2017	Oedema in wrists	Patient	2	1	Wrists	Brazil	23	Male		IgM, PRNT	Local
Zonneveld et al., 2016	Arthritis	Patient		1	Not given	Suriname	61	Male	Hypertension	RT-PCR	Local
Díaz-Quiñonez et al., 2016	Arthritis	Patient		1	Not given	Mexico	26	Male		RT-PCR	Imported

PRNT: Plaque reduction neutralisation test; RT-PCR: Reverse transcription polymerase chain reaction.

## Supplementary Materials

The following are available online at https://www.mdpi.com/1999-4915/12/10/1137/s1, Table S1: Case Studies reporting joint signs/symptoms, Table S2: Population based studies, Table S3: Forest plot of comparison of prevalence of arthralgia vs arthritis, Table S4: Forest plot of prevalence of arthralgia among confirmed ZIKV cases (number of population-based studies: 40), Table S5: Forest plot of prevalence of joint symptoms according to study designs of population-based studies, Table S6: Forest plot of prevalence of joint symptoms according to number of confirmed ZIKV cases (sample size), Table S7: Forest plot of prevalence of joint symptoms according to immune status, Table S8: Forest plot of prevalence of joint symptoms during outbreaks.
