# Supplementary material for: Zika Virus and Arthritis/Arthralgia: A Systematic Review and Meta-Analysis
Source: Viruses. 2020 Oct 7;12(10):1137. doi: 10.3390/v12101137 (PMC7599580; doi:10.3390/v12101137)
Supplement: Supplementary file 1 [file viruses-12-01137-s001.pdf]

Table S1. Case Studies reporting joint signs/symptoms

Authors	Country reported	No. of confirmed Zika cases	No. of cases reporting joint manifestations	Affected joint	Arthralgia duration (days)	Arthritis duration	Age (arthralgia/arthritis +ve patients)	Sex	Co-morbities	Lab confirmation by	Reference
Acosta-Reyes et al., 2017	Colombia	2	1		14		24	Female		rRT-PCR	[1]
Arsuaga et al., 2016	Spain	2	2			7, 5	53, 51	1 Male & 1 Female		RT-PCR	[2]
Aspahan et al., 2019	Brazil	1	1				35	Male	dengue fever 18 months prior (confirmed by serology at the time)	Zika PCR	[3]
Bachiller-Luque et al., 2016	Spain	1	1	Fingers, right knee, ankle and elbow		5	49	Male	irritable bowel syndrome, hypertension and dyslipidemia	RT-PCR	[4]
Bhatnagar et al., 2017	United States, Brazil, Columbia	32	9					9 Female		RT-PCR	[5]
Brust et al., 2014	United States	1	1		2		35	Female	otherwise healthy	IgM, PRNT	[6]

Calleri et al.., 2016	Italy	2	2		7,4		29, 31	1 Male, 1 Female		IgM/IgG and neutralisation	[7]
Candelo et al.., 2019	Colombia	2	1				25	Female		RT-PCR	[8]
Cardona-Cardona et al., 2016	Colombia	1	1		2		55	Female	previously healthy	RT-PCR	[9]
Cassuto et al..., 2018	France	1	1		5			Male		qRT-PCR,IgG and IgM	[10]
Cavalcanti et al.., 2017	Brazil	4	3	Arthralgia-ankle and knee, arthritis-fingers and toes	5,7,5	arthritis-3days	42,28,33	All Female		Real-time reverse transcriptase PCR RT-PCR	[11]
Chang et al.., 2018	Colombia	18	13				Inconclusive	Inconclusive		Neutralization assay	[12]
Chen et al.., 2017	United States	1	1				23	Female		RT-PCR	[13]
Colavita et al.., 2018	Italy	1	1		7		Not given	Female	rheumatoid arthritis	genome sequencing	[14]
Cosano-Quero et al.., 2018	Spain	3	1	Small joint			46	Male		PCR	[15]
Davidson, 2016	United States	2	2				20s	Female, Male		rRT-PCR	[16]
de Oliveira et al.., 2018	Brazil	1	1		2		50	Female		RT- PCR, sequencing	[17]
Derrington et al.., 2016	United States	1	1	Wrists, knees, ankles	4		44	Male		RT-PCR, IgM	[18]
Díaz-Quiñonez et al.., 2016	Mexico	1	1				26	Male		RT-PCR, sequencing	[19]
D'Ortenzio et al.., 2016	France	2	1		7		24	Female	previously healthy	RT-PCR	[20]

do Rosario et al.., 2016	Brazil	2	1		5	22	Male		IgM, PRNT	[21]
Duijster et al.., 2016	Netherlands	18	13			61, 31, 60, 33, 46, 47, 61, 54, 56, 61, 60, 54, 40	3 Males & 10femal es		RT-PCR	[22]
Edupuganti et al.., 2017	United States	1	1	Hips and knees	3 and recurre nt 14	60	Male	presumed nonalcoholic steatohepatiti s, thrombocytop enia of unknown etiology, hypertension, obesity, and obstructive sleep apnea	rRT-PCR	[23]
Estofolete et al.., 2016	Brazil	13	5			23, 41, 49, 67, 19	Female, Male, Female, Male, Female		ZIKV RT-PCR	[24]
Fabrizius et al.., 2016	United States	1	1	Both hands and Fingers		44	Male	without significant past medical history	RT-PCR	[25]
Fontes et al.., 2016	Brazil	1	1		12	51	Female		Confirmed in a panel of viruses at a reference lab	[26]

								but method not given	
Gaskell et al.., 2017	United Kingdom	1	1	10	45	Male	previously healthy	rRT-PCR, IgM and IgG	[27]
Goorhuis et al.., 2016	Netherlands	5	5		60, 40, 54, 47, 53	3 Female, 2 Males		PCR	[28]
Harrower et al.., 2016	New Zealand	2	2		51, 53	Male, Female		RT-PCR, IgM and IgG	[29]
Ho et al., 2018	Colombia	1	1		23	Female	no significant past medical history	IgM and IgG antibodies, and RT-PCR	[30]
Karam et al., 2017	Venezuela	1	1	6	22	Female	Hashimoto's thyroiditis	Ig M, PRNT	[31]
Khatib et al., 2019	Canada	2	1	3	40	Female	previously healthy	Ig M, RT-PCR	[32]
Khawar et al., 2017	United States	7	5	3,1,4	56, 28, ~~33~~, 17, 56, 48	2 Males And 2 Females		RT-PCR	[33]
Kodati et al., 2017	United States	1	1		26	Male	previously healthy	RT-PCR	[34]
Kulkarni et al., 2017	United States	1	1		42	Female	previously healthy	Serology and PRNT	[35]
Kutsuna et al., 2014	Japan	2	1	5	Mid 20s	Male	previously healthy	RT-PCR	[36]
Langerak et al., 2016	Suriname	3	1	7	40s	Male		IgG and Neutralisation	[37]
Mansuy et al., 2019	South America	1	1	few days	32	Male	immunocompetent white man	rRT-PCR	[38]

Maria et al., 2016	France	3	1		7		20s	Male		IgM, IgG and Neutralisatio n	[39]
Martinez et al., 2018	Brazil	1	1		5		42	Female	previously healthy	PCR	[40]
Meaney-Delman et al., 2016a	United States	4	2				20s &30s	2 Females		RT-PCR, Ig G & Ig M, PRNT	[41]
Meaney-Delman et al., 2016b	USA	4	3					3 Female		RT-PCR and immunohisto chemical staining	[42]
Medina et al., 2016	Honduras	1	1				62	Male		PRNT	[43]
Merle et al., 2017	Martinique	2	1				50s	Female		RT-PCR	[44]
Molko et al., 2017	New Caledonia	2	1		7		45	Male	previously healthy	RT-PCR	[45]
Moulin et al., 2016	Swizterland	1	1	Wrists and interphalange al joints			29	Female		RT-PCR	[46]
Neri et al., 2018	Brazil	1	1				38	Female		IgM, RT-PCR	[47]
Nicastri et al., 2016	Italy	3	1			6	74	Male		IgM, IgG, Neutralisatio n	[48]
Oliveira et al., 2018	Brazil	4	3	Case 1: Arthralgia on ankles and edema of fingers, Case 2: diffuse arthralgia	Case 1:3, Case 2:5	Case1: 3 days, Case3: 5 weeks	32, 33, 68	2 Females, 1 Male		qRT-PCR	[49]
Mondolfi et al., 2018	Venezuela	1	1	Small joint	5		68	Female	past medical history was unremarkable	RT-PCR, IgM	[50]

Paniz-Mondolfi et al., 2018	Venezuela	1	1	Distal small joint	3		15	Female	no significant past medical history	IgM & RT-PCR	[51]
Parke et al., 2016	United States	1	1	Knees and shoulders	10		64	Male	insignificant medical history	PRNT	[52]
Passos et al., 2017	Brazil	21	3				18, 25, 26		Three patients with no underlying conditions	RT-PCR	[53]
Penot et al., 2017	France	5	4		17,4	>10, >10	28, 36, 30, 39	4 Females	case 3: no relevant medical history, case 4: a substituted hypothyroidism and a mild, non-treated rheumatoid polyarthritis	PCR	[54]
Perkasa et al., 2016	Indonesia	1	1	Elbow and knee	4		27	Male		RT-PCR and sequencing	[55]
Piorkowski et al., 2016	Martinique	1	1				54	Female		RT-PCR	[56]
Pohl et al., 2018	Germany	2	2	Knee	Both cases 7		57, 53	Male, Female		Seroconversion and exclusion of dengue and chikungunya	[57]
Pokrovskiy et al., 2016	Russia	1	1				36	Female		RT-PCR	[58]

Ramos-Rossy et al., 2018	Puerto Rico	1	1	Diffuse arthralgia	7	80	Male	hypertension, diabetes mellitus,hyperlipidemia, peripheral vascular disease, chronic kidney disease stage III, and one coronary artery bypass graft	RT-PCR	[59]
Rozé et al., 2016	Martinique	2	2		45	Young adult & late 70s	Not Given	previously healthy	RT-PCR	[60]
Sanín-Blair et al., 2017	Colombia	3	2			30,16	2 Females		PCR	[61]
Sezen et al., 2018	Turkey	1	1			29	Male	otherwise healthy	RT-PCR	[62]
Soares et al., 2018	Brazil	1	1			47	Female	unremarkable medical history	IgM, IgG, RT-PCR	[63]
Summers et al., 2015	United States	1	1		9	48	Male	previously healthy	ZIKV IgG & IgM and exclusion of Dengue and WNV	[64]
Tappe et al., 2015	Germany	1	1	Wrists, palms and fingers	5	45	Female		IgG, IgM and viral	[65]

										neutralizatio n	
van der Eijk et al., 2016	Netherlands	1	1	Both wrists and the left knee	8		31-year-old	Female	medical history was uneventful	rRT-PCR	[66]
Valiant et al., 2018	United States	2	2		8		30, 28	Female, Male		RT-PCR, Serology	[67]
Vilibic-Cavlek et al., 2017	Croatia	1	1		7		Late 20s	Female	previously healthy	IgM/IgG, PRNT	[68]
Vinhaes et al., 2017	Brazil	1	1	Ankles and wrists	15	2	23	Male		IgM, PRNT	[69]
Wæhre et al., 2014	Norway	1	1				31	Female	previously healthy	RT-PCR, genome sequencing	[70]
Waggoner et al., 2017	United States	1	1		4		45	Female	mitral valve prolapses, irregular menses, and a congenital single kidney	RT-PCR, Focus reduction neutralizatio n test (FRNT)	[71]
Walker et al., 2018	United States	21	3				Not given	3 Females		IgM and RT-PCR, PRNT	[72]
Wright et al., 2019	Canada	1	1	Wrists and ankles			34	Female		PCR	[73]
Zammarchi et al., 2015	Italy	1	1				Early30s	Female		RT-PCR	[74]
Zea-Vera et al., 2017	Colombia	1	1		2		30	Female	immune thrombocytop enia (ITP)	RT-PCR	[75]
Zé-Zé et al., 2016	Portugal	4	3	Cases 1 & 2: hands, wrists and ankles,	>11 days		61, 59,62	1male & 2 Females	cases 1 and 2- no past medical history, case	RT-PCR/ IgG/IgM and exclusion of other viruses	[76]

				Case 3: discrete				3-with a past medical history of systemic lupus erythematosus, fibromyalgia, cardiomyopathy and hyperuricemia		
Zonneveld et al., 2016	Suriname	3	2			61, 59	2 Males	Case 1: hypertension, Case 2: chronic obstructive pulmonary disease	RT-PCR, sequencing	[77]
Zucker et al., 2017	United States	1	1		5	Adolescent	Not Given	medical history included mild depression	RT-PCR	[78]

Table S2. Population based studies

Authors	Country reported	Study design	Time (study period)	No. of cases with +ve lab diagnosis	No. of cases reporting arthralgia/ arthritis	Lab confirmation by	Reference
Adhikari et al., 2017	USA	Cohort study (prospective)	March 14 and Oct. 1, 2016	5	2	rRT-PCR, IgM, Serology and PRNT testing	[79]
Alva-Urcia et al., 2017	Peru	Cross-sectional	January 2016 to March 2016	7	3	RT-PCR	[80]
Ankrah et al., 2019	Ghana	Surveillance	December 2016 and November 2017	33	24	IgG/IgM, PRNT	[81]
Araúz et al., 2016	Guna Yala region of eastern Panama	Epidemiological study	November 27, 2015–January 22, 2016	50	20	Real-time reverse transcription PCR (rRT-PCR)	[82]
Armstrong et al., 2016	US	Surveillance	1 January 2015 to 26 February 2016	115	76	RT-PCR, IgM, Neutralising Abs	[83]
Barros et al., 2018	Goiânia, Goiás, Brazil	Case-control study	January–May 2016	36	15	RT-PCR	[84]
Bôtto-Menezes et al., 2019	Brazil	Cohort study	July 2017- until mid-2020	10	6	Zika rRT-PCR	[85]
Bozza et al., 2019	Brazil	Cohort study	Outbreak 2016	15	12	rRT-PCR	[86]
Brasil et al., 2016a	Rio de Janeiro	Cohort study	September 2015 through May 2016	130	81	RT-PCR	[87]
Brasil et al., 2016b	Brazil	Prospective syndromic surveillance study	January to July 2015	119	75	RT-PCR	[88]

Brooks et al., 2017	Roatán, Honduras	Cross-sectional survey	September 2015–July 2016	79	71	RT-PCR	[89]
Burger-Calderon et al., 2018	Nicaragua	Screening and surveillance	August 31 to October 21 2016	14	6	rRT-PCR, IgM	[90]
Carvalho et al., 2019	Rio de Janeiro (Brazil)	Cross-sectional	2015 to 2018	45	24	RT-qPCR	[91]
Cerbino-Neto et al., 2017	Rio de Janeiro, Brazil	Retrospective evaluation of electronic medical records and surveillance reports	April 28–June 8, 2015	57	Arthralgia: 33 (arthritis: 13)	rRT-PCR	[92]
Chow et al., 2017	Singapore	Cohort study	August 26–September 5, 2016	149	34	RT-PCR	[93]
Conners et al., 2018	New York City	Cohort study (retrospective)	January 1, 2016 to June 30, 2017	107	20	RT-PCR, Serology and PRNT	[94]
Crespillo-Andújar at al, 2020	Spain	Cohort study (prospective)	January 2016 to January 2017	25	14	RT-PCR or serology+ PRNT, IgM and IgG	[95]
Daudens-Vaysse et al., 2016	Martinique Island and French Polynesia	Surveillance	24 November 2015 to 20 January 2016	500	328	RT-PCR or seroneutralisation	[96]
de Laval et al., 2016	French Guiana	Systematic screening		8	3	rRT-PCR, IgM, or Neutralising Abs	[97]
de Laval et al., 2018	French Guiana	Longitudinal cohort survey	January – October 2016	49	26	RT-PCR	[98]
del Carpio-Orantes et al., 2019	Mexico	Descriptive, retrospective, and longitudinal study	1 August to 30 November 2016	25	25	RT-PCR	[99]
Duffy et al., 2009	Micronesia	Reviewed medical records and conducted prospective surveillance (49 confirmed cases: clinical data available only for 31 people)	April 1 through July 31, 2007	31	20	RT-PCR/IgM & PRNT	[100]
Flamand et al., 2017	French Guiana	Surveillance	1 February to 1 June 2016	573	56	RT-PCR, IgM, Neutralising Abs	[101]

Garcell et al., 2020	Cuba	Cross-sectional descriptive study	June 1st to October 31st, 2017	279	183	RNA in serum or urine-qualitative PCR	[102]
Garza-González et al., 2017	Mexico	Surveillance	September 13 to November 25 2016	99	45	rRT-PCR	[103]
Gregianini et al., 2017	Brazil	Epidemiological study	Between 2014 and 2016	41	17 (arthritis 8%)	RT-PCR	[104]
Griffin et al., 2017	Miami-Dade County, Florida	Surveillance	October 1, 2015, and March 29, 2017	33	7	RT-PCR/ MAC-ELISA for IgM & PRNT	[105]
Guerbois et al.,2016	southern Mexico	Epidemiological study	30 Nov to 18 December 2015	25	21	RT-PCR, PRNT and ELISA	[106]
Halai et al., 2017	Rio de Janeiro	Cohort study (prospective)	September 2015 to May 2016	130	81	PCR	[107]
Haque et al., 2016	Mexico	Surveillance	October 2015 and January 2016	84	67 (arthritis- 17)	rRT-PCR	[108]
Hennessey et al., 2016	US	Surveillance	2010 to 2014	11	7	RT-PCR, IgM, PRNT	[109]
Huits et al., 2017	Belgium	Cohort study (prospective)	February 2016 to May 2017	15	5	RT-PCR or neutralisation	[110]
Kam et al., 2017	Brazil	Cohort study (prospective)	Between February and August 2016	6	2	rRT-PCR, ZIKV NS1-specific ELISA	[111]
Katip et al., 2017	Chiang Mai, Thailand	Epidemiological analysis of retrospectively collected clinical data	June to Nov. 2016	19	7	RT-PCR	[112]
Lozier et al., 2018	Puerto Rico	Surveillance	16 September–27 October 2016	58	38 (arthritis-24)	RT-PCR, IgM	[113]

Millet et al., 2017	Barcelona, Spain	Cross-sectional population-based study, observational	January 1st and December 2016	44	26	IgG, IgM and PCR	[114]
Musso et al., 2017	French Polynesia	Hospital-based study; (Confirmed cases 210, clinical data only in 57)	October 2013 to March 2014	57	27	RT-PCR	[115]
Ng et al., 2018	Singapore	Cohort study (prospective)	26 August to 5 September 2016	40	15	rRT-PCR	[116]
Peña et al., 2019	Dominican Republic	Cross-sectional	January 2016–April 2017	225	108(mentioned as Arthritis/arthralgia)	IgM, serology and RT-PCR	[117]
Phan et al., 2019	southern Viet Nam	Surveillance	2016	214	66	rRT–PCR	[118]
Queiroz et al., 2019	Brazil	Case-control study	May 2015 to November 2016	12	11	RT-qPCR	[119]
Ramacciotti et al., 2019	Brazil	Surveillance & case reports	2016-2017	31	20	rRT-PCR	[120]
Read et al., 2018	Puerto Rico	Surveillance	January to December 2016	351	129	RT-PCR and IgM	[121]
Rodó et al., 2019	Barcelona (Catalonia, Spain)	Cross-sectional survey	January 2016 and September 2017	9	3	RT-PCR or IgM, IgG, Neutralization	[122]
Romer et al., 2019	Mexico	Cohort study (prospective)	July 1, 2016–august 31, 2017	36	15 (arthritis 07)	rRT-PCR	[123]
Sahly HM et al., 2019	United States	Cohort study, observational	Between July 13, 2016, and September 19, 2017	45	37 (arthritis 18)	RT-PCR, Neutralisation Abs	[124]
Sánchez-Carbonel et al., 2018	Piura, Peru	Cross-sectional, descriptive	May to August 2016	39	32	rRT-PCR	[125]

Silva et al., 2019	Salvador, Brazil	Surveillance	September 2014 - July, 2016	13	7 (swollen joints 04)	RT-PCR	[126]
Singh et al., 2019	Brazil	Cohort study (prospective)	July 2016 to October 2017	9	5	RT-PCR, IgM, IgG and/or neutralization serology	[127]
Thomas et al., 2016	Puerto Rico	Surveillance	November 23, 2015–January 28, 2016	30	22	RT- PCR or IgM	[128]
Tozetto-Mendoza et al.,2019	Brazil	Hospital-based study	January and September 2016	94	62 (articular edema 25/78)	RT-PCR, IgM & IgG	[129]
Uncini et al., 2018	Cucuta, Colombia	Cohort study	October 2015 to July 2016	20	15	Serology and plaque reduction neutralization test (PRNT90)	[130]
Vasquez et al., 2018	Paris, France	Epidemiological study	Jan 2009-dec.2016	36	28	RT-PCR	[131]
Vega et al.,2019	Santa Luzia, Minas Gerais, Brazil	Longitudinal study	EW01 to EW52 of 2016	12	1	qRT-PCR	[132]
Vieira et al., 2019	Brazil	Epidemiological study	First half of 2017	7	4	rRT-PCR	[133]
Villamil-Gómez et al., 2016	Colombia	Cohort study	Jan-16	28	11	RT-PCR	[134]
Vroon et al., 2017	Suriname	Cross-sectional		21	9	RT-PCR	[135]
Yoon et al., 2017	Korea	Epidemiological study	March to October 2016	14	4	RT-PCR	[136]
Zanluca et al., 2015	Natal, Brazil	Epidemiological study	Early 2015	8	7 (periarticular swelling 6/8)	RT-PCR and sequencing	[137]

PRNT: Plaque reduction neutralization test; rRT-PCR: Real-time reverse transcription polymerase chain reaction; quantitative Real-time reverse transcription PCR: qRT-PCR

Table S3: Forest plot of comparison of prevalence of arthralgia vs arthritis

Study or Subgroup	Arthritis Events	Arthritis Total	Arthralgia Events	Arthralgia Total	Weight	Odds Ratio M-H, Random, 95% CI
Cerbino-Neto et al, 2016	13	57	33	57	14.0%	0.21 [0.10, 0.48]
Gregianini et al, 2017	3	41	18	41	8.0%	0.10 [0.03, 0.38]
Haque et al, 2016	17	84	67	84	15.0%	0.06 [0.03, 0.14]
Lozier et al, 2018	24	58	38	58	15.0%	0.37 [0.18, 0.79]
Romer et al, 2019	7	36	15	36	10.6%	0.34 [0.12, 0.97]
Sahly et al, 2019	18	45	37	45	11.8%	0.14 [0.05, 0.38]
Silva et al, 2018	4	13	7	13	6.0%	0.38 [0.08, 1.90]
Tozetto-Mendoza et al, 2019	25	78	62	94	17.0%	0.24 [0.13, 0.46]
Zanluca et al, 2015	6	8	7	8	2.6%	0.43 [0.03, 5.98]
**Total (95% CI)**		**420**		**436**	**100.0%**	**0.20 [0.13, 0.31]**
Total events	117		284			

Heterogeneity: Tau² = 0.20; Chi² = 15.04, df = 8 (P = 0.06); I² = 47%
Test for overall effect: Z = 7.06 (P < 0.00001)

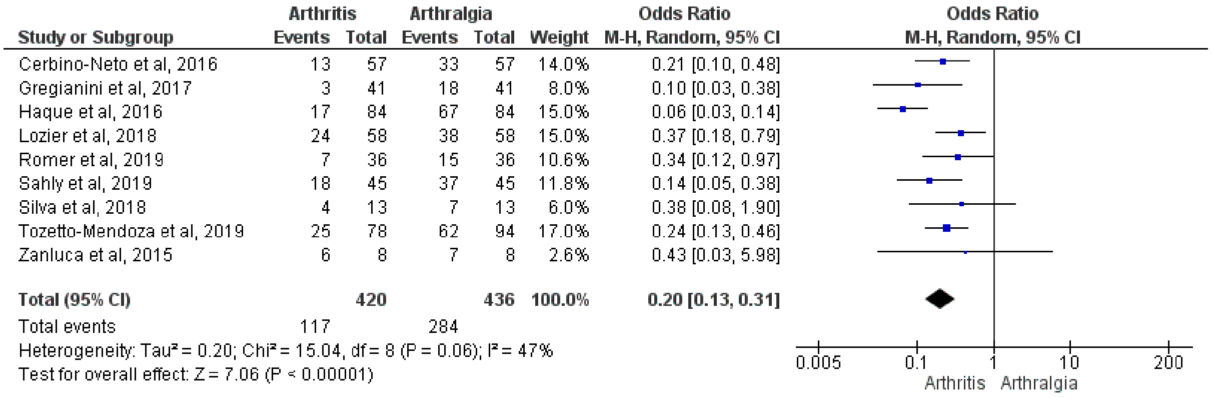

Table S4: Forest plot of prevalence of arthralgia among confirmed ZIKV cases (number of population-based studies: 40)

Study or Subgroup	Prevalence	SE	Weight	Prevalence IV, Random, 95% CI
Adhikari et al, 2017	6	18.8269	1.7%	6.00 [-30.90, 42.90]
Alva-Urcia et al, 2017	42.9	16.3779	1.9%	42.90 [10.80, 75.00]
Araúz et al, 2016	40	7.0409	2.6%	40.00 [26.20, 53.80]
Armstrong et al, 2016	66.1	4.0766	2.8%	66.10 [58.11, 74.09]
Barros et al, 2018	41.7	8.2144	2.5%	41.70 [25.60, 57.80]
Bozza et al, 2019	80	6.6328	2.7%	80.00 [67.00, 93.00]
Brasil et al, 2016p	60.4	4.0307	2.8%	60.40 [52.50, 68.30]
Brooks et al, 2019	89.9	2.5	2.9%	89.90 [85.00, 94.80]
Burger-Calderon et al, 2018	30.3	8.6736	2.5%	30.30 [13.30, 47.30]
Bôtto-Menezes et al, 2019	60	11.8267	2.3%	60.00 [36.82, 83.18]
Carvalho et al, 2019	53.3	7.0409	2.6%	53.30 [39.50, 67.10]
Chow et al, 2017	22.8	3.7756	2.8%	22.80 [15.40, 30.20]
Conners et al, 2018	18.7	4.2858	2.8%	18.70 [10.30, 27.10]
Crespillo-Andújar et al, 2020	56	8.8267	2.5%	56.00 [38.70, 73.30]
Daudens-Vaysse et al, 2016	66.5	3.1123	2.8%	66.50 [60.40, 72.60]
de Laval et al, 2016	27.3	14.9493	2.0%	27.30 [-2.00, 56.60]
del Carpio-Orantes et al, 2018	100	6.796	2.6%	100.00 [86.68, 113.32]
Flamand et al, 2017	42.1	4.3368	2.8%	42.10 [33.60, 50.60]
Garcell et al, 2019	65.6	2.7041	2.9%	65.60 [60.30, 70.90]
Garza-González et al, 2017	45.5	5.0001	2.8%	45.50 [35.70, 55.30]
Griffin et al, 2017	21.2	8.4695	2.5%	21.20 [4.60, 37.80]
Guerbois et al, 2016	84	4.898	2.8%	84.00 [74.40, 93.60]
Hennessey et al, 2016	63.6	10.8165	2.3%	63.60 [42.40, 84.80]
Huits et al, 2017	33.3	12.7553	2.2%	33.30 [8.30, 58.30]
Kam et al ,2017	33.3	18.7248	1.7%	33.30 [-3.40, 70.00]
Katip and Wientong, 2017	36.8	11.3267	2.3%	36.80 [14.60, 59.00]
Millet et al, 2019	59.1	6.7348	2.7%	59.10 [45.90, 72.30]
Phan et al, 2019	57.5	3.2654	2.8%	57.50 [51.10, 63.90]
Queiroz et al, 2019	91.7	3.4695	2.8%	91.70 [84.90, 98.50]
Ramacciotti et al, 2019	64.5	7.3369	2.6%	64.50 [50.12, 78.88]
Read et al, 2018	36.8	2.6021	2.9%	36.80 [31.70, 41.90]
Rodó et al, 2019	33.3	15.9697	1.9%	33.30 [2.00, 64.60]
Singh et al, 2019	55.6	13.0104	2.2%	55.60 [30.10, 81.10]
Sánchez-Carbonel et al, 2019	82.1	4.5409	2.8%	82.10 [73.20, 91.00]
Thomas et al, 2016	73.3	6.3777	2.7%	73.30 [60.80, 85.80]
Uncini et al, 2018	75	7.0409	2.6%	75.00 [61.20, 88.80]
Vieira et al, 2019	57.1	13.8268	2.1%	57.10 [30.00, 84.20]
Villamil-Gómez et al, 2016	39.3	9.3369	2.5%	39.30 [21.00, 57.60]
Vroon et al, 2017	42.9	10.5104	2.4%	42.90 [22.30, 63.50]
Yoon et al, 2017	28.6	13.3166	2.1%	28.60 [2.50, 54.70]
**Total (95% CI)**			**100.0%**	**53.55 [46.14, 60.96]**

Heterogeneity: $Tau^2 = 494.06$; $Chi^2 = 721.35$, df = 39 ($P < 0.00001$); $I^2 = 95\%$
Test for overall effect: $Z = 14.16$ ($P < 0.00001$)

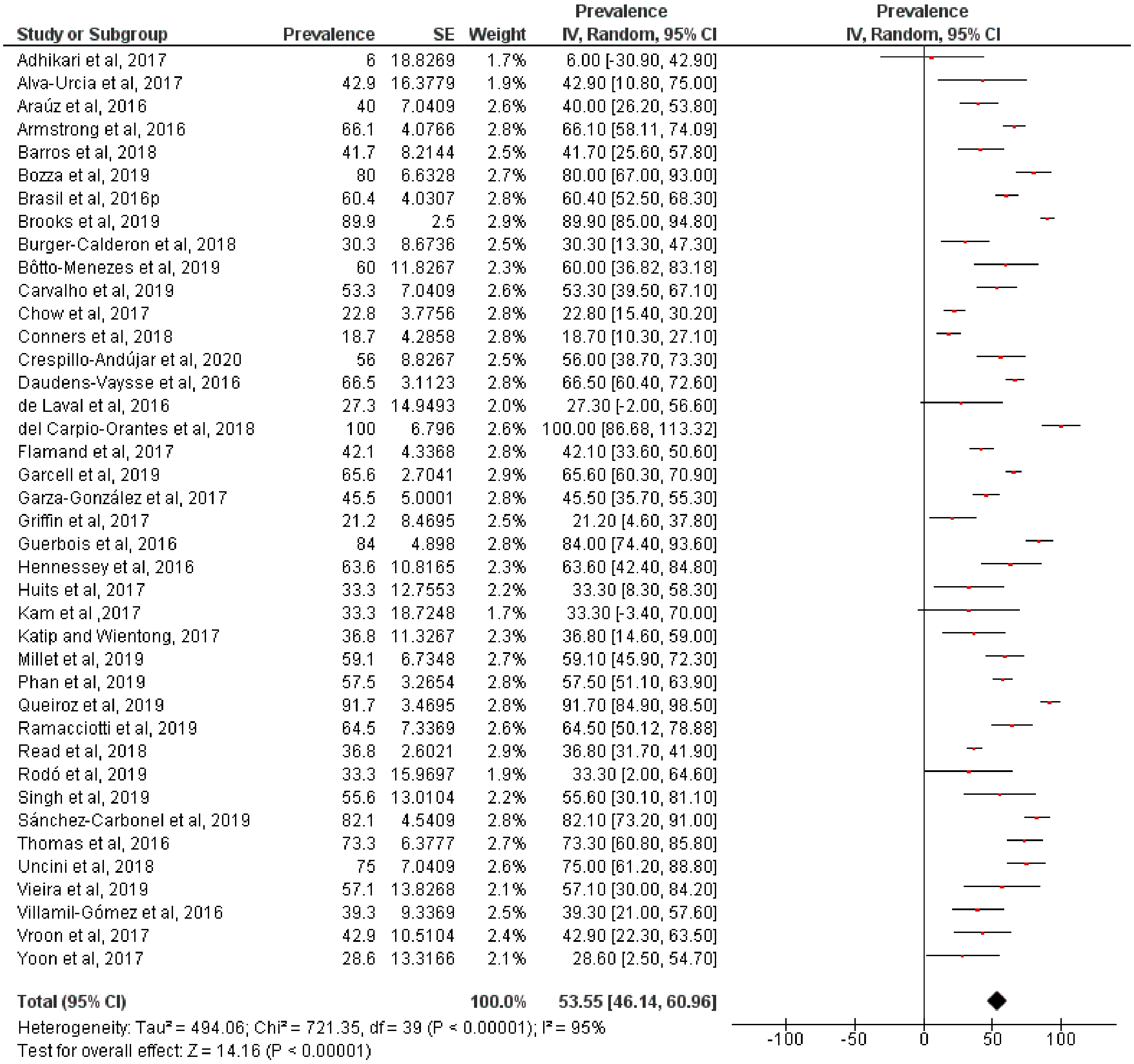

Table S5: Forest plot of prevalence of joint symptoms according to study designs of population-based studies

Study or Subgroup	Prevalence	SE	Weight	Prevalence IV, Fixed, 95% CI
**1.1.1 Epidemiological studies**				
Araúz et al, 2016	40	7.0409	0.9%	40.00 [26.20, 53.80]
Gregianini et al, 2017	43	8.143	0.7%	43.00 [27.04, 58.96]
Guerbois et al, 2016	84	4.898	1.9%	84.00 [74.40, 93.60]
Katip and Wientong, 2017	36.8	11.3267	0.4%	36.80 [14.60, 59.00]
Vasquez et al, 2018	77.8	5.3572	1.6%	77.80 [67.30, 88.30]
Vieira et al, 2019	57.1	13.8268	0.2%	57.10 [30.00, 84.20]
Yoon et al, 2017	28.6	13.3166	0.3%	28.60 [2.50, 54.70]
Zanluca et al, 2015	87.5	5.2552	1.6%	87.50 [77.20, 97.80]
**Subtotal (95% CI)**			**7.5%**	**69.50 [64.70, 74.30]**
Heterogeneity: Chi² = 69.61, df = 7 (P < 0.00001); I² = 90%				
Test for overall effect: Z = 28.37 (P < 0.00001)				
**1.1.2 Cohort studies**				
Adhikari et al, 2017	40	18.8269	0.1%	40.00 [3.10, 76.90]
Barros et al, 2018	41.7	8.2144	0.7%	41.70 [25.60, 57.80]
Bozza et al, 2019	80	6.6328	1.0%	80.00 [67.00, 93.00]
Brasil et al, 2016o	63	4.1838	2.6%	63.00 [54.80, 71.20]
Brasil et al, 2016p	60.4	4.0307	2.8%	60.40 [52.50, 68.30]
Bôtto-Menezes et al, 2019	60	11.8267	0.3%	60.00 [36.82, 83.18]
Chow et al, 2017	22.8	3.7756	3.2%	22.80 [15.40, 30.20]
Conners et al, 2018	18.7	4.2858	2.5%	18.70 [10.30, 27.10]
Crespillo-Andújar et al, 2020	56	8.8267	0.6%	56.00 [38.70, 73.30]
de Laval et al, 2018	53.06	6.7552	1.0%	53.06 [39.82, 66.30]
Halai et al, 2017	62.3	4.0307	2.8%	62.30 [54.40, 70.20]
Huits et al, 2017	33.3	12.7553	0.3%	33.30 [8.30, 58.30]
Kam et al ,2017	33.3	18.7248	0.1%	33.30 [-3.40, 70.00]
Ng et al, 2018	37.5	7.9083	0.7%	37.50 [22.00, 53.00]
Romer et al, 2019	41.7	8.2144	0.7%	41.70 [25.60, 57.80]
Sahly et al, 2019	82.2	4.3368	2.4%	82.20 [73.70, 90.70]
Singh et al, 2019	55.6	13.0104	0.3%	55.60 [30.10, 81.10]
Uncini et al, 2018	75	7.0409	0.9%	75.00 [61.20, 88.80]
Villamil-Gómez et al, 2016	39.3	9.3369	0.5%	39.30 [21.00, 57.60]
**Subtotal (95% CI)**			**23.3%**	**51.79 [49.07, 54.52]**
Heterogeneity: Chi² = 227.60, df = 18 (P < 0.00001); I² = 92%				
Test for overall effect: Z = 37.26 (P < 0.00001)				
**1.1.3 Screening and surveillance**				
Ankrah et al, 2019	72.7	6.2246	1.2%	72.70 [60.50, 84.90]
Armstrong et al, 2016	66.1	4.0766	2.7%	66.10 [58.11, 74.09]
Burger-Calderon et al, 2018	30.3	8.6736	0.6%	30.30 [13.30, 47.30]
Cerbino-Neto et al, 2016	57.9	6.0715	1.2%	57.90 [46.00, 69.80]
Daudens-Vaysse et al, 2016	66.5	3.1123	4.7%	66.50 [60.40, 72.60]
de Laval et al, 2016	27.3	14.9493	0.2%	27.30 [-2.00, 56.60]
Duffy et al, 2009	64.5	7.3471	0.8%	64.50 [50.10, 78.90]
Flamand et al, 2017	42.1	4.3368	2.4%	42.10 [33.60, 50.60]
Garza-González et al, 2017	45.5	5.0001	1.8%	45.50 [35.70, 55.30]
Griffin et al, 2017	39.4	8.6226	0.6%	39.40 [22.50, 56.30]
Haque et al, 2016	79.8	3.6735	3.3%	79.80 [72.60, 87.00]
Hennessey et al, 2016	63.6	10.8165	0.4%	63.60 [42.40, 84.80]
Lindsey et al, 2019	47.5	4.1838	2.6%	47.50 [39.30, 55.70]
Lozier et al, 2018	65.5	5.5613	1.5%	65.50 [54.60, 76.40]
Musso et al, 2017	12.9	18.1	0.1%	12.90 [-22.58, 48.38]
Phan et al, 2019	57.5	3.2654	4.2%	57.50 [51.10, 63.90]
Ramacciotti et al, 2019	64.5	7.3369	0.8%	64.50 [50.12, 78.88]
Read et al, 2018	36.8	2.6021	6.7%	36.80 [31.70, 41.90]
Silva et al, 2018	53.8	11.7349	0.3%	53.80 [30.80, 76.80]
Thomas et al, 2016	73.3	6.3777	1.1%	73.30 [60.80, 85.80]
Vroon et al, 2017	42.9	10.5104	0.4%	42.90 [22.30, 63.50]
**Subtotal (95% CI)**			**37.7%**	**55.70 [53.56, 57.85]**
Heterogeneity: Chi² = 177.06, df = 20 (P < 0.00001); I² = 89%				
Test for overall effect: Z = 50.91 (P < 0.00001)				
**1.1.4 Cross-sectional**				
Alva-Urcia et al, 2017	42.9	16.3779	0.2%	42.90 [10.80, 75.00]
Brooks et al, 2019	89.9	2.5	7.2%	89.90 [85.00, 94.80]
Carvalho et al, 2019	53.3	7.0409	0.9%	53.30 [39.50, 67.10]
Garcell et al, 2019	65.6	2.7041	6.2%	65.60 [60.30, 70.90]
Millet et al, 2019	59.1	6.7348	1.0%	59.10 [45.90, 72.30]
Peña et al, 2019	48	3.3164	4.1%	48.00 [41.50, 54.50]
Rodó et al, 2019	33.3	15.9697	0.2%	33.30 [2.00, 64.60]
Sánchez-Carbonel et al, 2019	82.1	4.5409	2.2%	82.10 [73.20, 91.00]
**Subtotal (95% CI)**			**21.9%**	**70.71 [67.90, 73.52]**
Heterogeneity: Chi² = 133.13, df = 7 (P < 0.00001); I² = 95%				
Test for overall effect: Z = 49.30 (P < 0.00001)				
**1.1.5 Hospital based**				
Brasil et al, 2016o	63	4.1838	2.6%	63.00 [54.80, 71.20]
Tozetto-Mendoza et al, 2019	66	4.4389	2.3%	66.00 [57.30, 74.70]
**Subtotal (95% CI)**			**4.9%**	**64.41 [58.44, 70.38]**
Heterogeneity: Chi² = 0.24, df = 1 (P = 0.62); I² = 0%				
Test for overall effect: Z = 21.16 (P < 0.00001)				
**1.1.6 Other**				
del Carpio-Orantes et al, 2018	100	6.796	1.0%	100.00 [86.68, 113.32]
Queiroz et al, 2019	91.7	3.4695	3.7%	91.70 [84.90, 98.50]
**Subtotal (95% CI)**			**4.7%**	**93.42 [87.36, 99.47]**
Heterogeneity: Chi² = 1.18, df = 1 (P = 0.28); I² = 15%				
Test for overall effect: Z = 30.23 (P < 0.00001)				
**Total (95% CI)**			**100.0%**	**61.32 [60.00, 62.64]**
Heterogeneity: Chi² = 845.15, df = 59 (P < 0.00001); I² = 93%				
Test for overall effect: Z = 91.33 (P < 0.00001)				
Test for subgroup differences: Chi² = 236.31, df = 5 (P < 0.00001), I² = 97.9%				

Prevalence
IV, Fixed, 95% CI

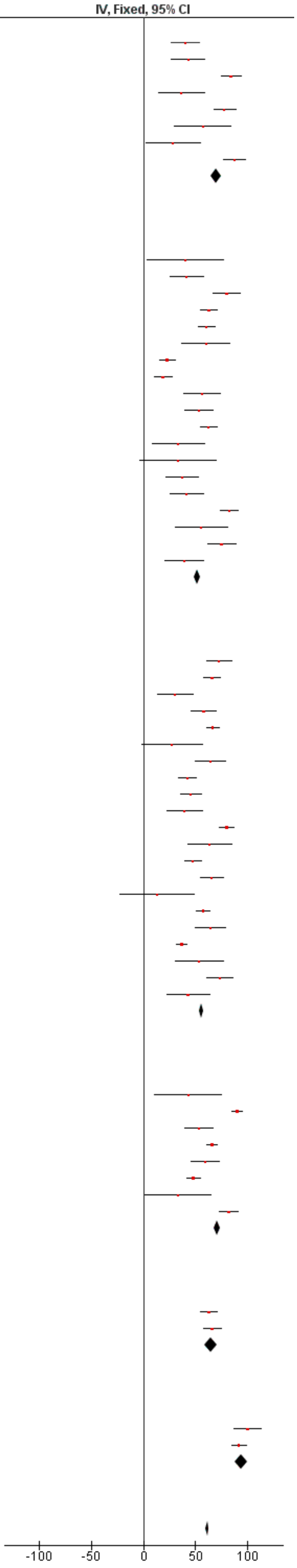

Table S6: Forest plot of prevalence of joint symptoms according to number of confirmed ZIKV cases (sample size)

Study or Subgroup	Prevalence	SE	Weight	Prevalence IV, Fixed, 95% CI
**1.4.1 0-20**				
Adhikari et al, 2017	40	18.8269	0.1%	40.00 [3.10, 76.90]
Alva-Urcia et al, 2017	42.9	16.3779	0.2%	42.90 [10.80, 75.00]
Bozza et al, 2019	80	6.6328	1.1%	80.00 [67.00, 93.00]
Burger-Calderon et al, 2018	30.3	8.6736	0.6%	30.30 [13.30, 47.30]
Bôtto-Menezes et al, 2019	60	11.8267	0.3%	60.00 [36.82, 83.18]
de Laval et al, 2016	27.3	14.9493	0.2%	27.30 [-2.00, 56.60]
Hennessey et al, 2016	63.6	10.8165	0.4%	63.60 [42.40, 84.80]
Huits et al, 2017	33.3	12.7553	0.3%	33.30 [8.30, 58.30]
Kam et al ,2017	33.3	18.7248	0.1%	33.30 [-3.40, 70.00]
Katip and Wientong, 2017	36.8	11.3267	0.4%	36.80 [14.60, 59.00]
Queiroz et al, 2019	91.7	3.4695	3.9%	91.70 [84.90, 98.50]
Rodó et al, 2019	33.3	15.9697	0.2%	33.30 [2.00, 64.60]
Silva et al, 2018	53.8	11.7349	0.3%	53.80 [30.80, 76.80]
Singh et al, 2019	55.6	13.0104	0.3%	55.60 [30.10, 81.10]
Uncini et al, 2018	75	7.0409	1.0%	75.00 [61.20, 88.80]
Vega et al, 2018	8.3	13.8268	0.2%	8.30 [-18.80, 35.40]
Vieira et al, 2019	57.1	13.8268	0.2%	57.10 [30.00, 84.20]
Yoon et al, 2017	28.6	13.3166	0.3%	28.60 [2.50, 54.70]
Zanluca et al, 2015	87.5	5.2552	1.7%	87.50 [77.20, 97.80]
**Subtotal (95% CI)**			**12.0%**	**70.69 [66.79, 74.60]**
Heterogeneity: Chi² = 147.89, df = 18 (P < 0.00001); I² = 88%				
Test for overall effect: Z = 35.50 (P < 0.00001)				
**1.4.2 21-50**				
Ankrah et al, 2019	72.7	6.2246	1.2%	72.70 [60.50, 84.90]
Araúz et al, 2016	40	7.0409	1.0%	40.00 [26.20, 53.80]
Barros et al, 2018	41.7	8.2144	0.7%	41.70 [25.60, 57.80]
Carvalho et al, 2019	53.3	7.0409	1.0%	53.30 [39.50, 67.10]
Crespillo-Andújar et al, 2020	56	8.8267	0.6%	56.00 [38.70, 73.30]
de Laval et al, 2018	53.06	6.7552	1.0%	53.06 [39.82, 66.30]
del Carpio-Orantes et al, 2018	100	6.796	1.0%	100.00 [86.68, 113.32]
Duffy et al, 2009	64.5	7.3471	0.9%	64.50 [50.10, 78.90]
Gregianini et al, 2017	43	8.143	0.7%	43.00 [27.04, 58.96]
Griffin et al, 2017	21.2	8.4695	0.7%	21.20 [4.60, 37.80]
Guerbois et al, 2016	84	4.898	2.0%	84.00 [74.40, 93.60]
Millet et al, 2019	59.1	6.7348	1.0%	59.10 [45.90, 72.30]
Ng et al, 2018	37.5	7.9083	0.8%	37.50 [22.00, 53.00]
Ramacciotti et al, 2019	64.5	7.3369	0.9%	64.50 [50.12, 78.88]
Romer et al, 2019	41.7	8.2144	0.7%	41.70 [25.60, 57.80]
Sahly et al, 2019	82.2	4.3368	2.5%	82.20 [73.70, 90.70]
Sánchez-Carbonel et al, 2019	82.1	4.5409	2.3%	82.10 [73.20, 91.00]
Thomas et al, 2016	73.3	6.3777	1.2%	73.30 [60.80, 85.80]
Vasquez et al, 2018	77.8	5.3572	1.7%	77.80 [67.30, 88.30]
Villamil-Gómez et al, 2016	39.3	9.3369	0.5%	39.30 [21.00, 57.60]
Vroon et al, 2017	42.9	10.5104	0.4%	42.90 [22.30, 63.50]
**Subtotal (95% CI)**			**22.7%**	**65.64 [62.81, 68.47]**
Heterogeneity: Chi² = 174.75, df = 20 (P < 0.00001); I² = 89%				
Test for overall effect: Z = 45.46 (P < 0.00001)				
**1.4.3 51-100**				
Brooks et al, 2019	89.9	2.5	7.6%	89.90 [85.00, 94.80]
Cerbino-Neto et al, 2016	57.9	6.0715	1.3%	57.90 [46.00, 69.80]
Garza-González et al, 2017	45.5	5.0001	1.9%	45.50 [35.70, 55.30]
Haque et al, 2016	79.8	3.6735	3.5%	79.80 [72.60, 87.00]
Lozier et al, 2018	65.5	5.5613	1.5%	65.50 [54.60, 76.40]
Musso et al, 2017	12.9	18.1	0.1%	12.90 [-22.58, 48.38]
Tozetto-Mendoza et al, 2019	66	4.4389	2.4%	66.00 [57.30, 74.70]
**Subtotal (95% CI)**			**18.4%**	**75.37 [72.22, 78.52]**
Heterogeneity: Chi² = 98.72, df = 6 (P < 0.00001); I² = 94%				
Test for overall effect: Z = 46.91 (P < 0.00001)				
**1.4.4 101-200**				
Armstrong et al, 2016	66.1	4.0766	2.9%	66.10 [58.11, 74.09]
Brasil et al, 2016o	63	4.1838	2.7%	63.00 [54.80, 71.20]
Brasil et al, 2016p	60.4	4.0307	2.9%	60.40 [52.50, 68.30]
Chow et al, 2017	22.8	3.7756	3.3%	22.80 [15.40, 30.20]
Conners et al, 2018	18.7	4.2858	2.6%	18.70 [10.30, 27.10]
Halai et al, 2017	62.3	4.0307	2.9%	62.30 [54.40, 70.20]
**Subtotal (95% CI)**			**17.3%**	**48.62 [45.38, 51.86]**
Heterogeneity: Chi² = 145.76, df = 5 (P < 0.00001); I² = 97%				
Test for overall effect: Z = 29.37 (P < 0.00001)				
**1.4.6 >200**				
Daudens-Vaysse et al, 2016	66.5	3.1123	4.9%	66.50 [60.40, 72.60]
Flamand et al, 2017	42.1	4.3368	2.5%	42.10 [33.60, 50.60]
Garcell et al, 2019	65.6	2.7041	6.5%	65.60 [60.30, 70.90]
Peña et al, 2019	48	3.3164	4.3%	48.00 [41.50, 54.50]
Phan et al, 2019	57.5	3.2654	4.4%	57.50 [51.10, 63.90]
Read et al, 2018	36.8	2.6021	7.0%	36.80 [31.70, 41.90]
**Subtotal (95% CI)**			**29.7%**	**53.18 [50.70, 55.66]**
Heterogeneity: Chi² = 89.76, df = 5 (P < 0.00001); I² = 94%				
Test for overall effect: Z = 42.06 (P < 0.00001)				
**Total (95% CI)**			**100.0%**	**61.39 [60.04, 62.74]**
Heterogeneity: Chi² = 864.74, df = 58 (P < 0.00001); I² = 93%				
Test for overall effect: Z = 89.17 (P < 0.00001)				
Test for subgroup differences: Chi² = 207.87, df = 4 (P < 0.00001), I² = 98.1%				

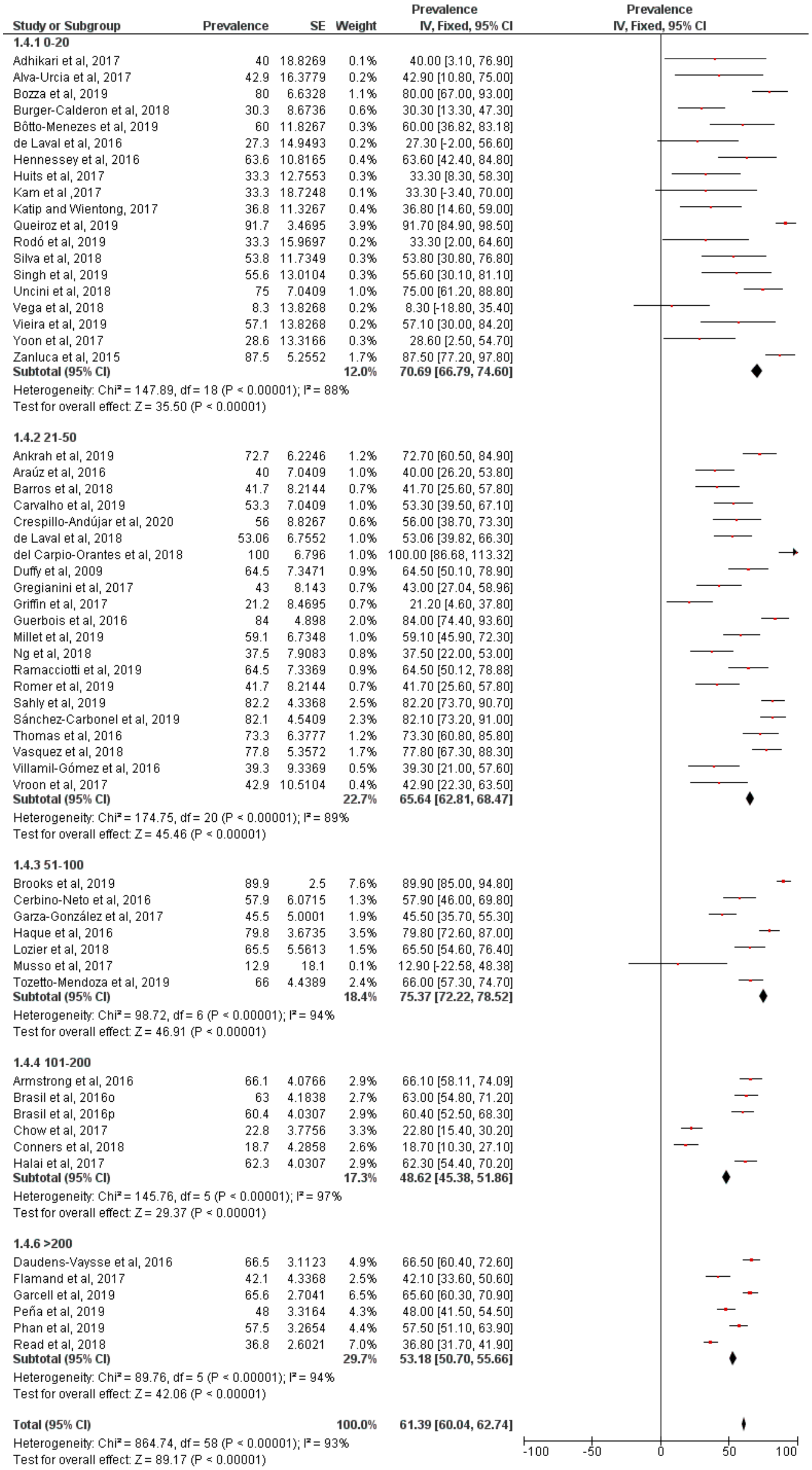

Table S7: Forest plot of prevalence of joint symptoms according to immune status

Study or Subgroup	Prevalence	SE	Weight	Prevalence IV, Fixed, 95% CI
**1.6.1 Pregnancy related**				
Adhikari et al, 2017	40	18.8269	0.4%	40.00 [3.10, 76.90]
Brasil et al, 2016p	60.4	4.0307	9.4%	60.40 [52.50, 68.30]
Carvalho et al, 2019	53.3	7.0409	3.1%	53.30 [39.50, 67.10]
Conners et al, 2018	18.7	4.2858	8.3%	18.70 [10.30, 27.10]
Flamand et al, 2017	42.1	4.3368	8.1%	42.10 [33.60, 50.60]
Garza-González et al, 2017	45.5	5.0001	6.1%	45.50 [35.70, 55.30]
Gregianini et al, 2017	43	8.143	2.3%	43.00 [27.04, 58.96]
Halai et al, 2017	62.3	4.0307	9.4%	62.30 [54.40, 70.20]
Kam et al ,2017	33.3	18.7248	0.4%	33.30 [-3.40, 70.00]
Peña et al, 2019	48	3.3164	13.8%	48.00 [41.50, 54.50]
Rodó et al, 2019	33.3	15.9697	0.6%	33.30 [2.00, 64.60]
Romer et al, 2019	41.7	8.2144	2.3%	41.70 [25.60, 57.80]
Singh et al, 2019	55.6	13.0104	0.9%	55.60 [30.10, 81.10]
Villamil-Gómez et al, 2016	39.3	9.3369	1.7%	39.30 [21.00, 57.60]
**Subtotal (95% CI)**			**66.8%**	**46.62 [43.66, 49.58]**
Heterogeneity: Chi² = 74.44, df = 13 (P < 0.00001); I² = 83%				
Test for overall effect: Z = 30.88 (P < 0.00001)				
**1.6.2 Children**				
Griffin et al, 2017	39.4	8.6226	2.0%	39.40 [22.50, 56.30]
Lindsey et al, 2019	47.5	4.1838	8.7%	47.50 [39.30, 55.70]
Read et al, 2018	36.8	2.6021	22.5%	36.80 [31.70, 41.90]
**Subtotal (95% CI)**			**33.2%**	**39.76 [35.57, 43.96]**
Heterogeneity: Chi² = 4.72, df = 2 (P = 0.09); I² = 58%				
Test for overall effect: Z = 18.58 (P < 0.00001)				
**Total (95% CI)**			**100.0%**	**44.34 [41.92, 46.76]**
Heterogeneity: Chi² = 86.02, df = 16 (P < 0.00001); I² = 81%				
Test for overall effect: Z = 35.94 (P < 0.00001)				
Test for subgroup differences: Chi² = 6.86, df = 1 (P = 0.009), I² = 85.4%				

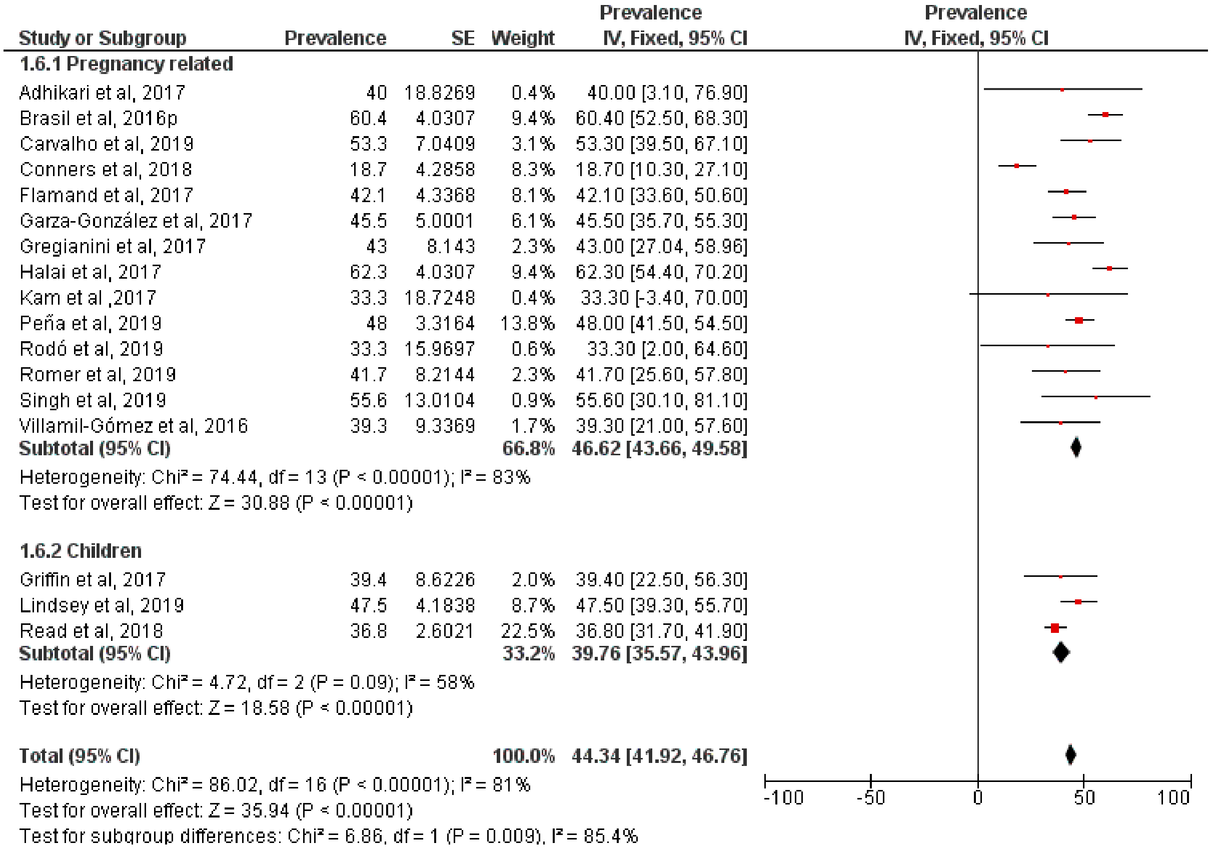

Table S8: Forest plot of prevalence of joint symptoms during outbreaks

Study or Subgroup	Prevalence	SE	Weight	Prevalence IV, Fixed, 95% CI
**1.5.1 Brazil outbreak 2015-2017**				
Brasil et al, 2016p	60.4	4.0307	12.4%	60.40 [52.50, 68.30]
Cerbino-Neto et al, 2016	57.9	6.0715	5.5%	57.90 [46.00, 69.80]
Tozetto-Mendoza et al, 2019	66	4.4389	10.2%	66.00 [57.30, 74.70]
Vroon et al, 2017	42.9	10.5104	1.8%	42.90 [22.30, 63.50]
**Subtotal (95% CI)**			**30.0%**	**60.79 [55.70, 65.88]**
Heterogeneity: Chi² = 4.51, df = 3 (P = 0.21); I² = 33%				
Test for overall effect: Z = 23.42 (P < 0.00001)				
**1.5.2 Puerto Rico**				
Lozier et al, 2018	65.5	5.5613	6.5%	65.50 [54.60, 76.40]
Thomas et al, 2016	73.3	6.3777	5.0%	73.30 [60.80, 85.80]
**Subtotal (95% CI)**			**11.5%**	**68.87 [60.65, 77.08]**
Heterogeneity: Chi² = 0.85, df = 1 (P = 0.36); I² = 0%				
Test for overall effect: Z = 16.43 (P < 0.00001)				
**1.5.3 Other American countries**				
Daudens-Vaysse et al, 2016	66.5	3.1123	20.8%	66.50 [60.40, 72.60]
Haque et al, 2016	79.8	3.6735	15.0%	79.80 [72.60, 87.00]
**Subtotal (95% CI)**			**35.8%**	**72.06 [67.40, 76.71]**
Heterogeneity: Chi² = 7.63, df = 1 (P = 0.006); I² = 87%				
Test for overall effect: Z = 30.34 (P < 0.00001)				
**1.5.4 SEA**				
Chow et al, 2017	22.8	3.7756	14.2%	22.80 [15.40, 30.20]
Katip and Wientong, 2017	36.8	11.3267	1.6%	36.80 [14.60, 59.00]
Ng et al, 2018	37.5	7.9083	3.2%	37.50 [22.00, 53.00]
**Subtotal (95% CI)**			**19.0%**	**26.46 [20.07, 32.86]**
Heterogeneity: Chi² = 3.72, df = 2 (P = 0.16); I² = 46%				
Test for overall effect: Z = 8.11 (P < 0.00001)				
**1.5.5 WPR**				
Duffy et al, 2009	64.5	7.3471	3.7%	64.50 [50.10, 78.90]
**Subtotal (95% CI)**			**3.7%**	**64.50 [50.10, 78.90]**
Heterogeneity: Not applicable				
Test for overall effect: Z = 8.78 (P < 0.00001)				
**Total (95% CI)**			**100.0%**	**59.38 [56.60, 62.17]**
Heterogeneity: Chi² = 152.90, df = 11 (P < 0.00001); I² = 93%				
Test for overall effect: Z = 41.79 (P < 0.00001)				
Test for subgroup differences: Chi² = 136.18, df = 4 (P < 0.00001), I² = 97.1%				

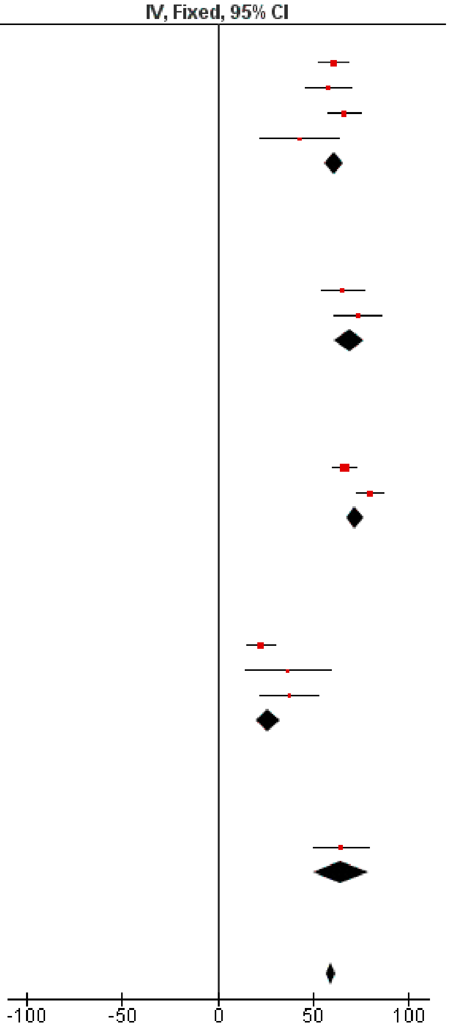

References

1. Acosta-Reyes, J.; Navarro, E.; Herrera, M.J.; Goenaga, E.; Ospina, M.L.; Parra, E.; Mercado, M.; Chaparro, P.; Beltran, M.; Gunturiz, M.L. Severe neurologic disorders in 2 fetuses with Zika virus infection, Colombia. *Emerging infectious diseases* **2017**, *23*, 982.
2. Arsuaga, M.; Bujalance, S.G.; Díaz-Menéndez, M.; Vázquez, A.; Arribas, J.R. Probable sexual transmission of Zika virus from a vasectomised man. *The Lancet Infectious diseases* **2016**, *16*, 1107.
3. Aspahan, M.C.; Leonhard, S.E.; Gomez, R.S.; da Silva, R.E.; da Silva, V.M.R.; Alvarenga, P.P.M.; Marinho, P.E.S.; Kroon, E.G.; Meira, F. Neuromyelitis optica spectrum disorder associated with Zika virus infection. *Neurology: Clinical Practice* **2019**, *9*, e1-e3.
4. Bachiller-Luque, P.; González, M.D.-G.; Álvarez-Manzanares, J.; Vázquez, A.; De Ory, F.; Fariñas, M.P.S.-S. First case of imported Zika virus infection in Spain. *Enfermedades infecciosas y microbiologia clinica* **2016**, *34*, 243-246.
5. Bhatnagar, J.; Rabeneck, D.B.; Martines, R.B.; Reagan-Steiner, S.; Ermias, Y.; Estetter, L.B.C.; Suzuki, T.; Ritter, J.; Keating, M.K.; Hale, G. Zika virus RNA replication and persistence in brain and placental tissue. *Emerging infectious diseases* **2017**, *23*, 405.
6. Brust, K.B.; Prince, W.S.; Fader, R.C. Trouble in paradise. *IDCases* **2014**, *1*, 95.
7. Calleri, G.; Burdino, E.; Bonora, S.; Raso, R.; Ghisetti, V.; Caramello, P. Zika virus infection in two travelers returning from an epidemic area to Italy, 2016: Algorithm for diagnosis and recommendations. *Travel medicine and infectious disease* **2016**, *14*, 506.
8. Candelo, E.; Caicedo, G.; Rosso, F.; Ballesteros, A.; Orrego, J.; Escobar, L.; Lapunzina, P.; Nevado, J.; Pachajoa, H. First report case with negative genetic study (array CGH, exome sequencing) in patients with vertical transmission of Zika virus infection and associated brain abnormalities. *The application of clinical genetics* **2019**, *12*, 141.
9. Cardona-Cardona, A.F.; Morales, A.J.R. Severe abdominal pain in a patient with Zika infection: a case in Risaralda, Colombia. *Journal of Infection and Public Health* **2016**, *9*, 372-373.
10. Cassuto, N.G.; Marras, G.; Jacomo, V.; Bouret, D. Persistence of Zika virus in gradient sperm preparation. *Journal of gynecology obstetrics and human reproduction* **2018**, *47*, 211.
11. Cavalcanti, M.G.; Cabral-Castro, M.J.; Gonçalves, J.L.S.; Santana, L.S.; Pimenta, E.S.; Peralta, J.M. Zika virus shedding in human milk during lactation: an unlikely source of infection? *International Journal of Infectious Diseases* **2017**, *57*, 70-72.
12. Chang, A.Y.; Lynch, R.; Martins, K.; Encinales, L.; Cadena, B.A.Á.; Pacheco, N.; Reid, S.P.; Lara, S.O.E.; González, T.H.J.; Mejia, C.S. Long-term clinical outcomes of Zika-associated Guillain-Barré syndrome. *Emerging microbes & infections* **2018**, *7*, 1-4.
13. Chen, L.; Hafeez, F.; Curry, C.L.; Elgart, G. Cutaneous eruption in a US woman with locally acquired Zika virus infection. *New England Journal of Medicine* **2017**, *376*, 400-401.
14. Colavita, F.; Musumeci, G.; Caglioti, C. Human osteoblast-like cells are permissive for Zika virus replication. *The Journal of rheumatology* **2018**, *45*, 443-443.
15. Cosano-Quero, A.; Velasco-Tirado, V.; Seco, M.P.S.; Manzanedo-Bueno, L.; Belhassen-García, M. Zika virus: cutaneous manifestations in 3 patients. *Actas Dermo-Sifiliográficas (English Edition)* **2018**, *109*, e13-e16.
16. Davidson, A.; Slavinski, S.; Komoto, K.; Rakeman, J.; Weiss, D. Suspected female-to-male sexual transmission of Zika virus - New York City, 2016. *MMWR Morb Mortal Wkly Rep* **2016**, *65*, 716-717, doi:10.15585/mmwr.mm6528e2.
17. de Oliveira, M.I.; Namiyama, G.M.; Cabral, G.B.; Ferreira, J.L.; Taniwaki, N.; Afonso, A.M.S.; Lima, I.R.; de Brigido, L.F.M. Isolation of infectious Zika virus from a urine sample cultured in SIRC cells from a patient suspected of having rubella virus. *Revista do Instituto de Medicina Tropical de São Paulo* **2018**, *60*.
18. Derrington, S.M.; Cellura, A.P.; McDermott, L.E.; Gubitosi, T.; Sonstegard, A.M.; Chen, S.; Garg, A. Mucocutaneous findings and course in an adult with Zika virus infection. *Jama Dermatology* **2016**, *152*, 691-693.
19. Díaz-Quiñonez, J.A.; Escobar-Escamilla, N.; Wong-Arambula, C.; Vazquez-Pichardo, M.; Torres-Longoria, B.; Lopez-Martinez, I.; Ruiz-Matus, C.; Kuri-Morales, P.; Ramírez-González, J.E. Asian genotype Zika virus detected in traveler returning to Mexico from Colombia, October 2015. *Emerging infectious diseases* **2016**, *22*, 937.
20. D'Ortenzio, E.; Matheron, S.; de Lamballerie, X.; Hubert, B.; Piorkowski, G.; Maquart, M.; Descamps, D.; Damond, F.; Yazdanpanah, Y.; Leparc-Goffart, I. Evidence of sexual transmission of Zika virus. *New England Journal of Medicine* **2016**, *374*, 2195-2198.

21. Do Rosário, M.S.; De Jesus, P.A.P.; Vasilakis, N.; Farias, D.S.; Novaes, M.A.C.; Rodrigues, S.G.; Martins, L.C.; da Costa, V.P.F.; Ko, A.I.; Alcântara, L.C.J. Guillain–Barre syndrome after zika virus infection in Brazil. *The American journal of tropical medicine and hygiene* **2016**, *95*, 1157-1160.
22. Duijster, J.W.; Goorhuis, A.; van Genderen, P.J.J.; Visser, L.G.; Koopmans, M.P.; Reimerink, J.H.; Grobusch, M.P.; van der Eijk, A.A.; van Den Kerkhof, J.H.C.T.; Reusken, C.B. Zika virus infection in 18 travellers returning from Surinam and the Dominican Republic, The Netherlands, November 2015–March 2016. *Infection* **2016**, *44*, 797-802.
23. Edupuganti, S.; Natrajan, M.S.; Rouphael, N.; Lai, L.; Xu, Y.; Feldhammer, M.; Hill, C.; Patel, S.M.; Johnson, S.J.; Bower, M. Biphasic Zika illness with rash and joint pain. In Proceedings of Open Forum Infectious Diseases.
24. Estofolete, C.F.; Terzian, A.C.B.; Parreira, R.; Esteves, A.; Hardman, L.; Greque, G.V.; Rahal, P.; Nogueira, M.L. Clinical and laboratory profile of Zika virus infection in dengue suspected patients: a case series. *Journal of Clinical Virology* **2016**, *81*, 25-30.
25. Fabrizius, R.G.; Anderson, K.; Hendel-Paterson, B.; Kaiser, R.M.; Maalim, S.; Walker, P.F. Guillain–Barre syndrome associated with Zika virus infection in a traveler returning from Guyana. *The American journal of tropical medicine and hygiene* **2016**, *95*, 1161-1165.
26. Fontes, C.A.P.; dos Santos, A.A.S.M.D.; Marchiori, E. Magnetic resonance imaging findings in Guillain-Barré syndrome caused by Zika virus infection. *Neuroradiology* **2016**, *58*, 837-838.
27. Gaskell, K.M.; Houlihan, C.; Nastouli, E.; Checkley, A.M. Persistent Zika virus detection in semen in a traveler returning to the United Kingdom from Brazil, 2016. *Emerging infectious diseases* **2017**, *23*, 137.
28. Goorhuis, A.; von Eije, K.J.; Douma, R.A.; Rijnberg, N.; van Vugt, M.; Stijnis, C.; Grobusch, M.P. Zika virus and the risk of imported infection in returned travelers: implications for clinical care. *Travel Medicine and Infectious Disease* **2016**, *14*, 13-15.
29. Harrower, J.; Kiedrzynski, T.; Baker, S.; Upton, A.; Rahnama, F.; Sherwood, J.; Huang, Q.S.; Todd, A.; Pulford, D. Sexual transmission of Zika virus and persistence in semen, New Zealand, 2016. *Emerging infectious diseases* **2016**, *22*, 1855.
30. Ho, C.-Y.; Castillo, N.; Encinales, L.; Porras, A.; Mendoza, A.R.; Lynch, R.; Nemirovsky, A.; Mantus, G.; DeBiasi, R.L.; Bethony, J.M. Second-trimester ultrasound and neuropathologic findings in congenital Zika virus infection. *The Pediatric infectious disease journal* **2018**, *37*, 1290.
31. Karam, E.; Giraldo, J.; Rodriguez, F.; Hernandez-Pereira, C.E.; Rodriguez-Morales, A.J.; Blohm, G.M.; Paniz-Mondolfi, A.E. Ocular flutter following Zika virus infection. *Journal of neurovirology* **2017**, *23*, 932-934.
32. Khatib, A.; Showler, A.J.; Kain, D.; Melvin, R.; Lecce, C.; Boggild, A.K. A diagnostic gap illuminated by a sexually-transmitted case of congenital Zika virus infection. *Travel medicine and infectious disease* **2019**, *27*, 117.
33. Khawar, W.; Bromberg, R.; Moor, M.; Lyubynska, N.; Mahmoudi, H. Seven cases of Zika virus infection in South Florida. *Cureus* **2017**, *9*.
34. Kodati, S.; Palmore, T.N.; Spellman, F.A.; Cunningham, D.; Weistrop, B.; Sen, H.N. Bilateral posterior uveitis associated with Zika virus infection. *The Lancet* **2017**, *389*, 125-126.
35. Kulkarni, S.A.; Strobelt, E.; Sargsyan, Z. Capillary fragility in Zika virus infection. *The American journal of medicine* **2017**, *130*, e59.
36. Kutsuna, S.; Kato, Y.; Takasaki, T.; Moi, M.L.; Kotaki, A.; Uemura, H.; Matono, T.; Fujiya, Y.; Mawatari, M.; Takeshita, N. Two cases of Zika fever imported from French Polynesia to Japan, December 2013 to January 2014. *Eurosurveillance* **2014**, *19*, 20683.
37. Langerak, T.; Yang, H.; Baptista, M.; Doornekamp, L.; Kerkman, T.; Codrington, J.; Roosblad, J.; Vreden, S.G.S.; De Bruin, E.; Mögling, R. Zika virus infection and Guillain–Barré syndrome in three patients from Suriname. *Frontiers in neurology* **2016**, *7*, 233.
38. Mansuy, J.-M.; El Costa, H.; Gouilly, J.; Mengelle, C.; Pasquier, C.; Martin-Blondel, G.; Izopet, J.; Jabrane-Ferrat, N. Peripheral plasma and semen cytokine response to Zika virus in humans. *Emerging infectious diseases* **2019**, *25*, 823.
39. Maria, A.T.; Maquart, M.; Makinson, A.; Flusin, O.; Segondy, M.; Leparc-Goffart, I.; Le Moing, V.; Foulongne, V. Zika virus infections in three travellers returning from South America and the Caribbean respectively, to Montpellier, France, December 2015 to January 2016. *Eurosurveillance* **2016**, *21*, 30131.
40. Martinez, A.R.M.; Costa, M.C.M.; Novaes, M.A.C.; Lima, H.C.; Nucci, A.; C., F.J.M. A novel phenotype Of Zika virus-related neurological disease: Sensory neuronopathy. *Muscle & Nerve* **2018**, *57*, E100-E101.

41. Meaney-Delman, D.; Oduyebo, T.; Polen, K.N.D.; White, J.L.; Bingham, A.M.; Slavinski, S.A.; Heberlein-Larson, L.; St George, K.; Rakeman, J.L.; Hills, S. Prolonged detection of Zika virus RNA in pregnant women. *Obstetrics & Gynecology* **2016**, *128*, 724-730.
42. Meaney-Delman, D.; Hills, S.L.; Williams, C.; Galang, R.R.; Iyengar, P.; Hennenfent, A.K.; Rabe, I.B.; Panella, A.; Oduyebo, T.; Honein, M.A. Zika virus infection among US pregnant travelers—August 2015–February 2016. *Morbidity and Mortality Weekly Report* **2016**, *65*, 211-214.
43. Medina, M.T.; England, J.D.; Lorenzana, I.; Medina-Montoya, M.; Alvarado, D.; De Bastos, M.; Fontiveros, S.; Sierra, M.; Contreras, F. Zika virus associated with sensory polyneuropathy. *Journal of the neurological sciences* **2016**, *369*, 271-272.
44. Merle, H.; Najioullah, F.; Chassery, M.; Césaire, R.; Hage, R. Zika-related bilateral hypertensive anterior acute uveitis. *Jama Ophthalmology* **2017**, *135*, 284-285.
45. Molko, N.; Simon, O.; Guyon, D.; Biron, A.; Dupont-Rouzeyrol, M.; Gourinat, A.-C. Zika virus infection and myasthenia gravis: report of 2 cases. *Neurology* **2017**, *88*, 1097-1098.
46. Moulin, E.; Selby, K.; Cherpillod, P.; Kaiser, L.; Boillat-Blanco, N. Simultaneous outbreaks of dengue, chikungunya and Zika virus infections: diagnosis challenge in a returning traveller with nonspecific febrile illness. *New microbes and new infections* **2016**, *11*, 6-7.
47. Neri, V.C.; Xavier, M.F.; Barros, P.O.; Bento, C.M.; Marignier, R.; Alvarenga, R.P. Case report: acute transverse myelitis after Zika virus infection. *The American journal of tropical medicine and hygiene* **2018**, *99*, 1419-1421.
48. Nicastri, E.; Pisapia, R.; Corpolongo, A.; Fusco, F.M.; Cicalini, S.; Scognamiglio, P.; Castilletti, C.; Bordi, L.; Di Caro, A.; Capobianchi, M.R. Three cases of Zika virus imported in Italy: need for a clinical awareness and evidence-based knowledge. *BMC infectious diseases* **2016**, *16*, 669.
49. Oliveira, D.B.L.; Durigon, G.S.; Mendes, É.A.; Ladner, J.T.; Andreata-Santos, R.; Araujo, D.B.; Botosso, V.F.; Paola, N.D.; Neto, D.F.L.; Cunha, M.P. Persistence and intra-host genetic evolution of Zika virus infection in symptomatic adults: a special view in the male reproductive system. *Viruses* **2018**, *10*, 615.
50. Mondolfi, A.E.P.; Peres, P.M.H.; Blohm, G.; Marquez, M.; Mendoza, A.M.; Hernandez-Pereira, C.E.; Escalona, M.A.; Colatosti, A.L.; DeArocha, J.R.; Morales, A.J.R. Generalized pustular psoriasis triggered by Zika virus infection. *Clinical and experimental dermatology* **2018**, *43*, 171-174.
51. Paniz-Mondolfi, A.E.; Giraldo, J.; Rodríguez-Morales, A.J.; Pacheco, O.; Lombó-Lucero, G.Y.; Plaza, J.D.; Adami-Teppa, F.J.; Carrillo, A.; Hernandez-Pereira, C.E.; Blohm, G.M. Alice in Wonderland syndrome: a novel neurological presentation of Zika virus infection. *Journal of NeuroVirology* **2018**, *24*, 660-663.
52. Parke, D.W.; Almeida, D.R.P.; Albini, T.A.; Ventura, C.V.; Berrocal, A.M.; Mittra, R.A. Serologically confirmed Zika-related unilateral acute maculopathy in an adult. *Ophthalmology* **2016**, *123*, 2432-2433.
53. Passos, S.R.L.; dos Santos, M.A.B.; Cerbino-Neto, J.; Buonora, S.N.; Souza, T.M.L.; de Oliveira, R.V.C.; Vizzoni, A.; Barbosa-Lima, G.; Vieira, Y.R.; de Lima, M.S. Detection of Zika virus in April 2013 patient samples, Rio de Janeiro, Brazil. *Emerging infectious diseases* **2017**, *23*, 2120.
54. Penot, P.; Balavoine, S.; Leplatois, A.; Brichler, S.; Leparc-Goffart, I.; Alloui, A.-C.; Flusin, O.; Guilleminot, J.; Amellou, M.; Molina, J.-M. Five cases of acute Zika virus infection in French women of reproductive age returning from Central and South America. *La Revue de medecine interne* **2017**, *38*, 547-550.
55. Perkasa, A.; Yudhaputri, F.; Haryanto, S.; Hayati, R.F.; Ma'roef, C.N.; Antonjaya, U.; Yohan, B.; Myint, K.S.A.; Ledermann, J.P.; Rosenberg, R. Isolation of Zika virus from febrile patient, Indonesia. *Emerging infectious diseases* **2016**, *22*, 924.
56. Piorkowski, G.; Richard, P.; Baronti, C.; Gallian, P.; Charrel, R.; Leparc-Goffart, I.; de Lamballerie, X. Complete coding sequence of Zika virus from Martinique outbreak in 2015. *New microbes and new infections* **2016**, *11*, 52-53.
57. Pohl, L.; Raulin, C.; Raulin, S. Zika virus in Germany: case report and possible routes of transmission. *JDDG: Journal der Deutschen Dermatologischen Gesellschaft* **2018**, *16*, 599-602.
58. Pokrovskiy, V.I.; Maleyev, V.V.; Krasnova, S.V.; Smetanina, S.V.; Vdovina, E.T.; Kotiv, S.I.; Karan, L.; Fedorova, M.V.; Grigor'eva, Y.E.; Valdokhina, A.V., et al.. The first case of Zika fever in Russia. *Infekcionnye bolezni* **2016**, *14*, 90-95, doi:10.20953/1729-9225-2016-1-90-95.
59. Ramos-Rossy, J.; Flores, J.; Otero-Domínguez, Y.; Torres-Palacios, J.; Rodríguez-Cintrón, W. Hypoxemic respiratory failure secondary to Zika virus infection. *Puerto Rico Health Sciences Journal* **2018**, *37*, 99-101.
60. Rozé, B.; Najioullah, F.; Signate, A.; Apetse, K.; Brouste, Y.; Gourgoudou, S.; Fagour, L.; Abel, S.; Hochedez, P.; Cesaire, R. Zika virus detection in cerebrospinal fluid from two patients with encephalopathy, Martinique, February 2016. *Eurosurveillance* **2016**, *21*, 30205.

61. Sanin-Blair, J.E.; Gutierrez-Marquez, C.; Herrera, D.A.; Vossough, A. Fetal magnetic resonance imaging findings in prenatal Zika virus infection. *Fetal Diagnosis and Therapy* **2017**, *42*, 153-157.
62. Sezen, A.İ.; Yıldırım, M.; Kültür, M.N.; Pehlivanoğlu, F.; Menemenlioğlu, D. Cases of Zika virus infection in Turkey: newly married couple returning from Cuba. *Mikrobiyoloji bulteni* **2018**, *52*, 308-315.
63. Soares, C.N.; Brasil, P.; Carrera, R.M.; Sequeira, P.; De Filippis, A.B.; Borges, V.A.; Theophilo, F.; Ellul, M.A.; Solomon, T. Fatal encephalitis associated with Zika virus infection in an adult. *Journal of Clinical Virology* **2016**, *83*, 63-65.
64. Summers, D.J.; Acosta, R.W.; Acosta, A.M. Zika virus in an American recreational traveler. *Journal of travel medicine* **2015**, *22*, 338-340.
65. Tappe, D.; Nachtigall, S.; Kapaun, A.; Schnitzler, P.; Günther, S.; Schmidt-Chanasit, J. Acute Zika virus infection after travel to Malaysian Borneo, September 2014. *Emerging infectious diseases* **2015**, *21*, 911.
66. van der Eijk, A.A.; van Genderen, P.J.; Verdijk, R.M.; Reusken, C.B.; Mögling, R.; van Kampen, J.J.A.; Widagdo, W.; Aron, G.I.; GeurtsvanKessel, C.H.; Pas, S.D. Miscarriage associated with Zika virus infection. *New England Journal of Medicine* **2016**, *375*, 1002-1004.
67. Valiant, W.G.; Lalani, T.; Yun, H.C.; Kunz, A.; Burgess, T.H.; Mattapallil, J.J. Human serum with high neutralizing antibody titers against both Zika and dengue virus shows delayed in vitro antibody-dependent enhancement of dengue virus infection. In Proceedings of Open forum infectious diseases; p. ofy151.
68. Vilibic-Cavlek, T.; Betica-Radic, L.; Venturi, G.; Fortuna, C.; Djuricic, S.; Salvia-Milos, A.; Tabain, I.; Barbic, L.; Stevanovic, V.; Listes, E. First detection of Zika virus infection in a Croatian traveler returning from Brazil, 2016. *The Journal of Infection in Developing Countries* **2017**, *11*, 662-667.
69. Vinhaes, E.S.; Santos, L.A.; Dias, L.; Andrade, N.A.; Bezerra, V.H.; De Carvalho, A.T.; De Moraes, L.; Henriques, D.F.; Azar, S.R.; Vasilakis, N. Transient hearing loss in adults associated with Zika virus infection. *Clinical Infectious Diseases* **2017**, *64*, 675-677.
70. Wæhre, T.; Maagard, A.; Tappe, D.; Cadar, D.; Schmidt-Chanasit, J. Zika virus infection after travel to Tahiti, December 2013. *Emerging infectious diseases* **2014**, *20*, 1412.
71. Waggoner, J.J.; Rouphael, N.; Xu, Y.; Natrajan, M.; Lai, L.; Patel, S.M.; Levit, R.D.; Edupuganti, S.; Mulligan, M.J. Pericarditis associated with acute Zika virus infection in a returning traveler. In Proceedings of Open Forum Infectious Diseases; p. ofx103.
72. Walker, C.L.; Merriam, A.A.; Ohuma, E.O.; Dighe, M.K.; Gale Jr, M.; Rajagopal, L.; Papageorghiou, A.T.; Gyamfi-Bannerman, C.; Waldorf, K.M.A. Femur-sparing pattern of abnormal fetal growth in pregnant women from New York City after maternal Zika virus infection. *American journal of obstetrics and gynecology* **2018**, *219*, 187. e181-187. e120.
73. Wright, J.K.; Castellani, L.; Lecce, C.; Khatib, A.; Bonta, M.; Boggild, A.K. Zika virus-associated aseptic meningitis and Guillain–Barre syndrome in a traveler returning from Latin America: A case report and mini-review. *Current infectious disease reports* **2019**, *21*, 1-9.
74. Zammarchi, L.; Stella, G.; Mantella, A.; Bartolozzi, D.; Tappe, D.; Günther, S.; Oestereich, L.; Cadar, D.; Muñoz-Fontela, C.; Bartoloni, A. Zika virus infections imported to Italy: clinical, immunological and virological findings, and public health implications. *Journal of Clinical Virology* **2015**, *63*, 32-35.
75. Zea-Vera, A.F.; Parra, B. Zika virus (ZIKV) infection related with immune thrombocytopenic purpura (ITP) exacerbation and antinuclear antibody positivity. *Lupus* **2017**, *26*, 890-892.
76. Ze-Ze, L.; Prata, M.B.; Teixeira, T.; Marques, N.; Mondragão, A.; Fernandes, R.; da Cunha, J.S.; Alves, M.J. Zika virus infections imported from Brazil to Portugal, 2015. *IDCases* **2016**, *4*, 46-49.
77. Zonneveld, R.; Roosblad, J.; van Staveren, J.W.; Wilschut, J.C.; Vreden, S.G.S.; Codrington, J. Three atypical lethal cases associated with acute Zika virus infection in Suriname. *IDCases* **2016**, *5*, 49-53.
78. Zucker, J.; Neu, N.; Chiriboga, C.A.; Hinton, V.J.; Leonardo, M.; Sheikh, A.; Thakur, K. Zika virus–associated cognitive impairment in adolescent, 2016. *Emerging infectious diseases* **2017**, *23*, 1047.
79. Adhikari, E.H.; Nelson, D.B.; Johnson, K.A.; Jacobs, S.; Rogers, V.L.; Roberts, S.W.; Sexton, T.; McIntire, D.D.; Casey, B.M. Infant outcomes among women with Zika virus infection during pregnancy: results of a large prenatal Zika screening program. *Am J Obstet Gynecol* **2017**, *216*, 292.e291-292.e298, doi:10.1016/j.ajog.2017.01.018.
80. Alva-Urcia, C.; Aguilar-Luis, M.A.; Palomares-Reyes, C.; Silva-Caso, W.; Suarez-Ognio, L.; Weilg, P.; Manrique, C.; Vasquez-Achaya, F.; Del Valle, L.J.; Del Valle-Mendoza, J. Emerging and reemerging arboviruses: A new threat in Eastern Peru. *PLoS One* **2017**, *12*, e0187897, doi:10.1371/journal.pone.0187897.

81. Ankrah, G.A.; Bonney, J.H.K.; Agbosu, E.E.; Pratt, D.; Adiku, T.K. Serological evidence of Zika virus infection in febrile patients at Greater Accra Regional Hospital, Accra Ghana. *BMC Res Notes* **2019**, *12*, 326, doi:10.1186/s13104-019-4371-4.
82. Araúz, D.; De Urriola, L.; Jones, J.; Castillo, M.; Martínez, A.; Murillo, E.; Troncoso, L.; Chen, M.; Abrego, L.; Armién, B., et al.. Febrile or exanthematous illness associated with Zika, dengue, and chikungunya viruses, Panama. *Emerg Infect Dis* **2016**, *22*, 1515-1517, doi:10.3201/eid2208.160292.
83. Armstrong, P.; Hennessey, M.; Adams, M.; Cherry, C.; Chiu, S.; Harrist, A.; Kwit, N.; Lewis, L.; McGuire, D.O.; Oduyebo, T., et al.. Travel-associated Zika virus disease cases among U.S. residents--United States, January 2015-February 2016. *MMWR Morb Mortal Wkly Rep* **2016**, *65*, 286-289, doi:10.15585/mmwr.mm6511e1.
84. Barros, J.B.S.; da Silva, P.A.N.; Koga, R.C.R.; Gonzalez-Dias, P.; Carmo Filho, J.R.; Nagib, P.R.A.; Coelho, V.; Nakaya, H.I.; Fonseca, S.G.; Pfrimer, I.A.H. Acute Zika virus infection in an endemic area shows modest proinflammatory systemic immunoactivation and Cytokine-symptom associations. *Front Immunol* **2018**, *9*, 821, doi:10.3389/fimmu.2018.00821.
85. Bôtto-Menezes, C.H.A.; Neto, A.M.; Calvet, G.A.; Kara, E.O.; Lacerda, M.V.G.; Castilho, M.D.C.; Ströher, U.; Antunes de Brito, C.A.; Modjarrad, K.; Broutet, N., et al.. Zika virus in rectal swab samples. *Emerg Infect Dis* **2019**, *25*, 951-954, doi:10.3201/eid2505.180904.
86. Bozza, F.A.; Moreira-Soto, A.; Rockstroh, A.; Fischer, C.; Nascimento, A.D.; Calheiros, A.S.; Drosten, C.; Bozza, P.T.; Souza, T.M.L.; Ulbert, S., et al.. Differential shedding and antibody kinetics of Zika and chikungunya viruses, Brazil. *Emerg Infect Dis* **2019**, *25*, 311-315, doi:10.3201/eid2502.180166.
87. Brasil, P.; Pereira, J.P., Jr.; Moreira, M.E.; Ribeiro Nogueira, R.M.; Damasceno, L.; Wakimoto, M.; Rabello, R.S.; Valderramos, S.G.; Halai, U.A.; Salles, T.S., et al.. Zika virus infection in pregnant women in Rio de Janeiro. *N Engl J Med* **2016**, *375*, 2321-2334, doi:10.1056/NEJMoa1602412.
88. Brasil, P.; Calvet, G.A.; Siqueira, A.M.; Wakimoto, M.; de Sequeira, P.C.; Nobre, A.; Quintana Mde, S.; Mendonça, M.C.; Lupi, O.; de Souza, R.V., et al.. Zika virus outbreak in Rio de Janeiro, Brazil: Clinical characterization, epidemiological and virological aspects. *PLoS Negl Trop Dis* **2016**, *10*, e0004636, doi:10.1371/journal.pntd.0004636.
89. Brooks, T.; Roy-Burman, A.; Tuholske, C.; Busch, M.P.; Bakkour, S.; Stone, M.; Linnen, J.M.; Gao, K.; Coleman, J.; Bloch, E.M. Real-time evolution of Zika virus disease outbreak, Roatán, Honduras. *Emerg Infect Dis* **2017**, *23*, 1360-1363, doi:10.3201/eid2308.161944.
90. Burger-Calderon, R.; Gonzalez, K.; Ojeda, S.; Zambrana, J.V.; Sanchez, N.; Cerpas Cruz, C.; Suazo Laguna, H.; Bustos, F.; Plazaola, M.; Lopez Mercado, B., et al.. Zika virus infection in Nicaraguan households. *PLoS Negl Trop Dis* **2018**, *12*, e0006518, doi:10.1371/journal.pntd.0006518.
91. Carvalho, F.R.; Medeiros, T.; Vianna, R.A.O.; Douglass-Jaimes, G.; Nunes, P.C.G.; Quintans, M.D.S.; Souza, C.F.; Cavalcanti, S.M.B.; Dos Santos, F.B.; Oliveira, S.A., et al.. Simultaneous circulation of arboviruses and other congenital infections in pregnant women in Rio de Janeiro, Brazil. *Acta Trop* **2019**, *192*, 49-54, doi:10.1016/j.actatropica.2019.01.020.
92. Cerbino-Neto, J.; Mesquita, E.C.; Souza, T.M.; Parreira, V.; Wittlin, B.B.; Durovni, B.; Lemos, M.C.; Vizzoni, A.; Bispo de Filippis, A.M.; Sampaio, S.A., et al.. Clinical manifestations of Zika virus infection, Rio de Janeiro, Brazil, 2015. *Emerg Infect Dis* **2016**, *22*, 1318-1320, doi:10.3201/eid2207.160375.
93. Chow, A.; Ho, H.; Win, M.K.; Leo, Y.S. Assessing sensitivity and specificity of surveillance case definitionsfor Zika virus disease. *Emerg Infect Dis* **2017**, *23*, 677-679, doi:10.3201/eid2304.161716.
94. Conners, E.E.; Lee, E.H.; Thompson, C.N.; McGibbon, E.; Rakeman, J.L.; Iwamoto, M.; Cooper, H.; Vora, N.M.; Limberger, R.J.; Fine, A.D., et al.. Zika virus infection among pregnant women and their neonates in New York City, January 2016-June 2017. *Obstet Gynecol* **2018**, *132*, 487-495, doi:10.1097/aog.0000000000002737.
95. Crespillo-Andújar, C.; Díaz-Menéndez, M.; Trigo, E.; Arsuaga, M.; De la Calle, F.; Lago, M.; de Guevara, M.C.L.; Barreiro, P.; Montero, D.; Garcia-Bujalance, S., et al.. Characteristics of Zika virus infection among international travelers: A prospective study from a Spanish referral unit. *Travel Med Infect Dis* **2020**, *33*, 101543, doi:10.1016/j.tmaid.2019.101543.
96. Daudens-Vaysse, E.; Ledrans, M.; Gay, N.; Ardillon, V.; Cassadou, S.; Najioullah, F.; Leparc-Goffart, I.; Rousset, D.; Herrmann, C.; Cesaire, R., et al.. Zika emergence in the French Territories of America and description of first confirmed cases of Zika virus infection on Martinique, November 2015 to February 2016. *Euro Surveill* **2016**, *21*, doi:10.2807/1560-7917.Es.2016.21.28.30285.
97. de Laval, F.; Matheus, S.; Maquart, M.; Yvrard, E.; Barthes, N.; Combes, C.; Rousset, D.; Leparc-Goffart, I.; Briolant, S. Prospective Zika virus disease cohort: systematic screening. *Lancet* **2016**, *388*, 868, doi:10.1016/s0140-6736(16)31429-5.

98. de Laval, F.; d'Aubigny, H.; Mathéus, S.; Labrousse, T.; Ensargueix, A.L.; Lorenzi, E.M.; Le Flem, F.X.; André, N.; Belleoud, D.; Leparc-Goffart, I., et al.. Evolution of symptoms and quality of life during Zika virus infection: A 1-year prospective cohort study. *J Clin Virol* **2018**, *109*, 57-62, doi:10.1016/j.jcv.2018.09.015.
99. Del Carpio-Orantes, L.; Rosas-Lozano, A.L.; García-Méndez, S. Zika virus infection in pregnant women in a General Hospital of Veracruz, Mexico. *J Matern Fetal Neonatal Med* **2019**, 10.1080/14767058.2019.1582627, 1-5, doi:10.1080/14767058.2019.1582627.
100. Duffy, M.R.; Chen, T.H.; Hancock, W.T.; Powers, A.M.; Kool, J.L.; Lanciotti, R.S.; Pretrick, M.; Marfel, M.; Holzbauer, S.; Dubray, C., et al.. Zika virus outbreak on Yap Island, Federated States of Micronesia. *N Engl J Med* **2009**, *360*, 2536-2543, doi:10.1056/NEJMoa0805715.
101. Flamand, C.; Fritzell, C.; Matheus, S.; Dueymes, M.; Carles, G.; Favre, A.; Enfissi, A.; Adde, A.; Demar, M.; Kazanji, M., et al.. The proportion of asymptomatic infections and spectrum of disease among pregnant women infected by Zika virus: systematic monitoring in French Guiana, 2016. *Euro Surveill* **2017**, *22*, doi:10.2807/1560-7917.Es.2017.22.44.17-00102.
102. Garcell, H.G.; García, F.G.; Nodal, M.R.; Lozano, A.R.; Díaz, C.R.P.; Valdés, A.G.; Alvarez, L.G. Clinical relevance of Zika symptoms in the context of a Zika Dengue epidemic. *J Infect Public Health* **2020**, *13*, 173-176, doi:10.1016/j.jiph.2019.07.006.
103. Garza-González, E.; Mendoza-Olazarán, S.; Roman-Campos, R.; Téllez-Marroquín, R.; Saldívar-Rodríguez, D.; Soria-López, J.A.; Guzman, A.; Flores-Treviño, S.; Camacho-Ortiz, A. Rapid spread of an ongoing outbreak of Zika virus disease in pregnant women in a Mexican hospital. *Braz J Infect Dis* **2017**, *21*, 554-556, doi:10.1016/j.bjid.2017.04.008.
104. Gregianini, T.S.; Ranieri, T.; Favreto, C.; Nunes, Z.M.A.; Tumioto Giannini, G.L.; Sanberg, N.D.; da Rosa, M.T.M.; da Veiga, A.B.G. Emerging arboviruses in Rio Grande do Sul, Brazil: Chikungunya and Zika outbreaks, 2014-2016. *Rev Med Virol* **2017**, *27*, doi:10.1002/rmv.1943.
105. Griffin, I.; Zhang, G.; Fernandez, D.; Cordero, C.; Logue, T.; White, S.L.; Llau, A.; Thomas, L.; Moore, E.; Noya-Chaveco, P., et al.. Epidemiology of pediatric Zika virus infections. *Pediatrics* **2017**, *140*, doi:10.1542/peds.2017-2044.
106. Guerbois, M.; Fernandez-Salas, I.; Azar, S.R.; Danis-Lozano, R.; Alpuche-Aranda, C.M.; Leal, G.; Garcia-Malo, I.R.; Diaz-Gonzalez, E.E.; Casas-Martinez, M.; Rossi, S.L., et al.. Outbreak of Zika virus infection, Chiapas State, Mexico, 2015, and first confirmed transmission by *Aedes aegypti* mosquitoes in the Americas. *J Infect Dis* **2016**, *214*, 1349-1356, doi:10.1093/infdis/jiw302.
107. Halai, U.A.; Nielsen-Saines, K.; Moreira, M.L.; de Sequeira, P.C.; Junior, J.P.P.; de Araujo Zin, A.; Cherry, J.; Gabaglia, C.R.; Gaw, S.L.; Adachi, K., et al.. Maternal Zika virus disease severity, virus load, prior dengue antibodies, and their relationship to birth outcomes. *Clin Infect Dis* **2017**, *65*, 877-883, doi:10.1093/cid/cix472.
108. Haque, U.; Ball, J.D.; Zhang, W.; Khan, M.M.H.; Treviño, C.J. Clinical and spatial features of Zika virus in Mexico. *Acta Trop* **2016**, *162*, 5-10, doi:10.1016/j.actatropica.2016.06.010.
109. Hennessey, M.J.; Fischer, M.; Panella, A.J.; Kosoy, O.I.; Laven, J.J.; Lanciotti, R.S.; Staples, J.E. Zika Virus disease in travelers returning to the United States, 2010-2014. *Am J Trop Med Hyg* **2016**, *95*, 212-215, doi:10.4269/ajtmh.16-0049.
110. Huits, R.; De Smet, B.; Ariën, K.K.; Van Esbroeck, M.; Bottieau, E.; Cnops, L. Zika virus in semen: a prospective cohort study of symptomatic travellers returning to Belgium. *Bull World Health Organ* **2017**, *95*, 802-809, doi:10.2471/blt.17.181370.
111. Kam, Y.W.; Leite, J.A.; Lum, F.M.; Tan, J.J.L.; Lee, B.; Judice, C.C.; Teixeira, D.A.T.; Andreata-Santos, R.; Vinolo, M.A.; Angerami, R., et al.. Specific biomarkers associated with neurological complications and congenital central nervous system abnormalities from Zika virus-infected patients in Brazil. *J Infect Dis* **2017**, *216*, 172-181, doi:10.1093/infdis/jix261.
112. Katip, W.; Wientong, P. Clinical characteristics and meteorological patterns of zika virus infection in Chiang Mai Province, Thailand. *The Southeast Asian journal of tropical medicine and public health* **2017**, *48*, 993-998.
113. Lozier, M.J.; Burke, R.M.; Lopez, J.; Acevedo, V.; Amador, M.; Read, J.S.; Jara, A.; Waterman, S.H.; Barrera, R.; Muñoz-Jordan, J., et al.. Differences in prevalence of symptomatic Zika virus infection, by age and sex-Puerto Rico, 2016. *J Infect Dis* **2018**, *217*, 1678-1689, doi:10.1093/infdis/jix630.
114. Millet, J.P.; Montalvo, T.; Bueno-Marí, R.; Romero-Tamarit, A.; Prats-Uribe, A.; Fernández, L.; Camprubí, E.; Del Baño, L.; Peracho, V.; Figuerola, J., et al.. Imported Zika virus in a European city: How to prevent local transmission? *Front Microbiol* **2017**, *8*, 1319, doi:10.3389/fmicb.2017.01319.

115. Musso, D.; Rouault, E.; Teissier, A.; Lanteri, M.C.; Zisou, K.; Broult, J.; Grange, E.; Nhan, T.X.; Aubry, M. Molecular detection of Zika virus in blood and RNA load determination during the French Polynesian outbreak. *J Med Virol* **2017**, *89*, 1505-1510, doi:10.1002/jmv.24735.
116. Ng, D.H.L.; Ho, H.J.; Chow, A.; Wong, J.; Kyaw, W.M.; Tan, A.; Chia, P.Y.; Choy, C.Y.; Tan, G.; Yeo, T.W., et al.. Correlation of clinical illness with viremia in Zika virus disease during an outbreak in Singapore. *BMC Infect Dis* **2018**, *18*, 301, doi:10.1186/s12879-018-3211-9.
117. Peña, F.; Pimentel, R.; Khosla, S.; Mehta, S.D.; Brito, M.O. Zika virus epidemic in pregnant women, Dominican Republic, 2016-2017. *Emerg Infect Dis* **2019**, *25*, 247-255, doi:10.3201/eid2502.181054.
118. Phan, L.T.; Luong, Q.C.; Do, T.H.H.; Chiu, C.H.; Cao, T.M.; Nguyen, T.T.T.; Diep, H.T.; Huynh, T.P.; Nguyen, D.T.; Le, N.H., et al.. Findings and lessons from establishing Zika virus surveillance in southern Viet Nam, 2016. *Western Pac Surveill Response J* **2019**, *10*, 22-30, doi:10.5365/wpsar.2018.9.2.014.
119. Queiroz, A.; Pinto, I.F.D.; Lima, M.; Giovanetti, M.; de Jesus, J.G.; Xavier, J.; Barreto, F.K.; Canuto, G.A.B.; do Amaral, H.R.; de Filippis, A.M.B., et al.. Lipidomic analysis reveals serum alteration of plasmalogens in patients infected with ZIKA virus. *Front Microbiol* **2019**, *10*, 753, doi:10.3389/fmicb.2019.00753.
120. Ramacciotti, E.; Agati, L.B.; Aguiar, V.C.R.; Wolosker, N.; Guerra, J.C.; de Almeida, R.P.; Alves, J.C.; Lopes, R.D.; Wakefield, T.W.; Comerota, A.J., et al.. Zika and chikungunya virus and risk for venous thromboembolism. *Clin Appl Thromb Hemost* **2019**, *25*, 1076029618821184, doi:10.1177/1076029618821184.
121. Read, J.S.; Torres-Velasquez, B.; Lorenzi, O.; Rivera Sanchez, A.; Torres-Torres, S.; Rivera, L.V.; Capre-Franceschi, S.M.; Garcia-Gubern, C.; Munoz-Jordan, J.; Santiago, G.A., et al.. Symptomatic Zika virus infection in infants, children, and adolescents living in Puerto Rico. *JAMA Pediatr* **2018**, *172*, 686-693, doi:10.1001/jamapediatrics.2018.0870.
122. Rodó, C.; Suy, A.; Sulleiro, E.; Soriano-Arandes, A.; Maiz, N.; García-Ruiz, I.; Arévalo, S.; Rando, A.; Anton, A.; Vázquez Méndez, É., et al.. Pregnancy outcomes after maternal Zika virus infection in a non-endemic region: prospective cohort study. *Clin Microbiol Infect* **2019**, *25*, 633.e635-633.e639, doi:10.1016/j.cmi.2019.02.008.
123. Romer, Y.; Valadez-Gonzalez, N.; Contreras-Capetillo, S.; Manrique-Saide, P.; Vazquez-Prokopec, G.; Pavia-Ruz, N. Zika virus infection in pregnant women, Yucatan, Mexico. *Emerg Infect Dis* **2019**, *25*, 1452-1460, doi:10.3201/eid2508.180915.
124. El Sahly, H.M.; Gorchakov, R.; Lai, L.; Natrajan, M.S.; Patel, S.M.; Atmar, R.L.; Keitel, W.A.; Hoft, D.F.; Barrett, J.; Bailey, J., et al.. Clinical,virologic, and immunologic characteristics of Zika virus infection in a cohort of US Patients: Prolonged RNA detection in whole blood. *Open Forum Infect Dis* **2019**, *6*, ofy352, doi:10.1093/ofid/ofy352.
125. Sánchez-Carbonel, J.; Tantaléan-Yépez, D.; Aguilar-Luis, M.A.; Silva-Caso, W.; Weilg, P.; Vásquez-Achaya, F.; Costa, L.; Martins-Luna, J.; Sandoval, I.; Del Valle-Mendoza, J. Identification of infection by Chikungunya, Zika, and Dengue in an area of the Peruvian coast. Molecular diagnosis and clinical characteristics. *BMC Res Notes* **2018**, *11*, 175, doi:10.1186/s13104-018-3290-0.
126. Silva, M.M.O.; Tauro, L.B.; Kikuti, M.; Anjos, R.O.; Santos, V.C.; Gonçalves, T.S.F.; Paploski, I.A.D.; Moreira, P.S.S.; Nascimento, L.C.J.; Campos, G.S., et al.. Concomitant transmission of dengue, chikungunya, and Zika viruses in Brazil: Clinical and epidemiological findings from surveillance for acute febrile illness. *Clin Infect Dis* **2019**, *69*, 1353-1359, doi:10.1093/cid/ciy1083.
127. Singh, T.; Lopez, C.A.; Giuberti, C.; Dennis, M.L.; Itell, H.L.; Heimsath, H.J.; Webster, H.S.; Roark, H.K.; Merçon de Vargas, P.R.; Hall, A., et al.. Efficient transplacental IgG transfer in women infected with Zika virus during pregnancy. *PLoS Negl Trop Dis* **2019**, *13*, e0007648, doi:10.1371/journal.pntd.0007648.
128. Thomas, D.L.; Sharp, T.M.; Torres, J.; Armstrong, P.A.; Munoz-Jordan, J.; Ryff, K.R.; Martinez-Quiñones, A.; Arias-Berríos, J.; Mayshack, M.; Garayalde, G.J., et al.. Local transmission of Zika virus--Puerto Rico, November 23, 2015-January 28, 2016. *MMWR Morb Mortal Wkly Rep* **2016**, *65*, 154-158, doi:10.15585/mmwr.mm6506e2.
129. Tozetto-Mendoza, T.R.; Avelino-Silva, V.I.; Fonseca, S.; Claro, I.M.; Paula, A.V.; Levin, A.S.; Sabino, E.C.; Mendes-Correa, M.C.; Figueiredo, W.M.; Felix, A.C., et al.. Zika virus infection among symptomatic patients from two healthcare centers in Sao Paulo State, Brazil: prevalence, clinical characteristics, viral detection in body fluids and serodynamics. *Rev Inst Med Trop Sao Paulo* **2019**, *61*, e19, doi:10.1590/s1678-9946201961019.
130. Uncini, A.; González-Bravo, D.C.; Acosta-Ampudia, Y.Y.; Ojeda, E.C.; Rodríguez, Y.; Monsalve, D.M.; Ramírez-Santana, C.; Vega, D.A.; Paipilla, D.; Torres, L., et al.. Clinical and nerve conduction features in Guillain-Barré syndrome associated with Zika virus infection in Cúcuta, Colombia. *Eur J Neurol* **2018**, *25*, 644-650, doi:10.1111/ene.13552.

131. Vasquez, V.; Haddad, E.; Perignon, A.; Jaureguiberry, S.; Brichler, S.; Leparc-Goffart, I.; Caumes, E. Dengue, chikungunya, and Zika virus infections imported to Paris between 2009 and 2016: Characteristics and correlation with outbreaks in the French overseas territories of Guadeloupe and Martinique. *Int J Infect Dis* **2018**, *72*, 34-39, doi:10.1016/j.ijid.2018.05.007.
132. Vega, F.L.R.; Bezerra, J.M.T.; Said, R.F.C.; Gama Neto, A.N.D.; Cotrim, E.C.; Mendez, D.; Amâncio, F.F.; Carneiro, M. Emergence of chikungunya and Zika in a municipality endemic to dengue, Santa Luzia, MG, Brazil, 2015-2017. *Rev Soc Bras Med Trop* **2019**, *52*, e20180347, doi:10.1590/0037-8682-0347-2018.
133. Vieira, D.S.; Zambenedetti, M.R.; Requião, L.; Borghetti, I.A.; Luna, L.K.S.; Santos, A.O.D.; Taborda, R.L.M.; Pereira, D.B.; Krieger, M.A.; Salcedo, J.M.V., et al.. Epidemiological profile of Zika, Dengue and Chikungunya virus infections identified by medical and molecular evaluations in Rondonia, Brazil. *Rev Inst Med Trop Sao Paulo* **2019**, *61*, e40, doi:10.1590/s1678-9946201961040.
134. Villamil-Gómez, W.E.; Mendoza-Guete, A.; Villalobos, E.; González-Arismendy, E.; Uribe-García, A.M.; Castellanos, J.E.; Rodríguez-Morales, A.J. Diagnosis, management and follow-up of pregnant women with Zika virus infection: A preliminary report of the ZIKERNCOL cohort study on Sincelejo, Colombia. *Travel Med Infect Dis* **2016**, *14*, 155-158, doi:10.1016/j.tmaid.2016.02.004.
135. Vroon, P.; Roosblad, J.; Poese, F.; Wilschut, J.; Codrington, J.; Vreden, S.; Zonneveld, R. Severity of acute Zika virus infection: A prospective emergency room surveillance study during the 2015-2016 outbreak in Suriname. *IDCases* **2017**, *10*, 117-121, doi:10.1016/j.idcr.2017.10.007.
136. Yoon, D.; Shin, S.H.; Jang, H.C.; Kim, E.S.; Song, E.H.; Moon, S.M.; Shin, S.Y.; Choe, P.G.; Sung, J.J.; Choi, E.H., et al.. Epidemiology and clinical characteristics of Zika virus infections imported into Korea from March to October 2016. *J Korean Med Sci* **2017**, *32*, 1440-1444, doi:10.3346/jkms.2017.32.9.1440.
137. Zanluca, C.; Melo, V.C.; Mosimann, A.L.; Santos, G.I.; Santos, C.N.; Luz, K. First report of autochthonous transmission of Zika virus in Brazil. *Mem Inst Oswaldo Cruz* **2015**, *110*, 569-572, doi:10.1590/0074-02760150192.
